# A National French Consensus on Gene List for the Diagnosis of Charcot–Marie–Tooth Disease and Related Disorders Using Next-Generation Sequencing

**DOI:** 10.3390/genes13020318

**Published:** 2022-02-09

**Authors:** Thibaut Benquey, Emmanuelle Pion, Mireille Cossée, Martin Krahn, Tanya Stojkovic, Aurélien Perrin, Mathieu Cerino, Annamaria Molon, Anne-Sophie Lia, Corinne Magdelaine, Bruno Francou, Anne Guiochon-Mantel, Marie-Claire Malinge, Eric Leguern, Nicolas Lévy, Shahram Attarian, Philippe Latour, Nathalie Bonello-Palot

**Affiliations:** 1 Service de Biochimie et Biologie Moléculaire Grand Est, Hospices Civils de Lyon, LBMMS, 69002 Lyon, France; Thibaut.benquey@chu-lyon.fr (T.B.); philippe.latour@chu-lyon.fr (P.L.); 2 Filnemus, CHRU Montpellier, 34093 Montpellier, France; emmanuelle.pion@ext.inserm.fr; 3 Laboratoire de Génétique Moléculaire, Centre Hospitalier Universitaire de Montpellier, 34093 Montpellier, France; mireille.cossee@inserm.fr; 4 PhyMedExp, Université de Montpellier, INSERM, CNRS, 34093 Montpellier, France; aurelien.perrin@ext.inserm.fr; 5 Marseille Medical Genetics, INSERM, UMR 1251, Aix-Marseille Université, 13005 Marseille, France; martin.krahn@ap-hm.fr (M.K.); mathieu.cerino@ap-hm.fr (M.C.); nicolas.levy@ap-hm.fr (N.L.); shahram.attarian@ap-hm.fr (S.A.); 6 Département de Génétique Médicale, APHM, Hôpital Timone Enfants, 13005 Marseille, France; 7 Institut de Myologie, Centre de Référence des Maladies Neuromusculaires Nord/Est/Ile de France, Hôpital Pitié-Salpêtrière, Sorbonne Université, 75013 Paris, France; stojkovich.tanya@aphp.fr; 8 Filnemus, APHM, 13005 Marseille, France; Annamaria.molon@ap-hm.fr; 9 Service de Biochimie et de Génétique Moléculaire, Centre de Biologie et de Recherche en Santé, CHU Limoges, 87042 Limoges, France; Anne-Sophie.LIA@unilim.fr (A.-S.L.); corinne.magdelaine87@gmail.com (C.M.); 10 Service de Génétique Moléculaire, Pharmacogénétique et Hormonologie, Centre Hospitalier Universitaire Bicêtre, Assistance Publique-Hôpitaux de Paris, 94270 Le Kremlin-Bicêtre, France; bruno.francou@aphp.fr (B.F.); anne.mantel@aphp.fr (A.G.-M.); 11 INSERM UMR1185, Faculté de médecine Paris Saclay, Université Paris-Saclay, 94270 Le Kremlin-Bicêtre, France; 12 Département de Biochimie Génétique, UF de Biologie Moléculaire, CHU d’Angers, 49100 Angers, France; mcmalinge@chu-angers.fr; 13 Centre de Génétique Moléculaire et Chromosomique, UF de Neurogénétique Moléculaire et Cellulaire APHP, 75651 Paris, France; eric.leguern@upmc.fr; 14 Reference Centres for Neuromuscular Diseases and ALS, Hôpital Timone Adultes, Assistance Publique Hôpitaux de Marseille, 13005 Marseille, France

**Keywords:** rare diseases, public health, Charcot–Marie–Tooth disease, next generation sequencing, consensus gene list

## Abstract

Next generation sequencing (NGS) is strategically used for genetic diagnosis in patients with Charcot–Marie–Tooth disease (CMT) and related disorders called non-syndromic inherited peripheral neuropathies (NSIPN) in this paper. With over 100 different CMT-associated genes involved and ongoing discoveries, an important interlaboratory diversity of gene panels exists at national and international levels. Here, we present the work of the French National Network for Rare Neuromuscular Diseases (FILNEMUS) genetic diagnosis section which coordinates the seven French diagnosis laboratories using NGS for peripheral neuropathies. This work aimed to establish a unique, simple and accurate gene classification based on literature evidence. In NSIPN, three subgroups were usually distinguished: (1) HMSN, Hereditary Motor Sensory Neuropathy, (2) dHMN, distal Hereditary Motor Neuropathy, and (3) HSAN, Hereditary Sensory Autonomic Neuropathy. First, we reported ClinGen evaluation, and second, for the genes not evaluated yet by ClinGen, we classified them as “definitive” if reported in at least two clinical publications and associated with one report of functional evidence, or “limited” otherwise. In total, we report a unique consensus gene list for NSIPN including the three subgroups with 93 genes definitive and 34 limited, which is a good rate for our gene’s panel for molecular diagnostic use.

## 1. Introduction

### 1.1. CMT Is a Heterogeneous Genetic Disease

Charcot–Marie–Tooth disease (CMT) encompasses Hereditary Motor and Sensory Neuropathies (HMSN). They represent the most frequent inherited peripheral neuropathy. Related disorders with overlapping clinical findings include distal Hereditary Motor Neuropathies (dHMN), Hereditary Sensory Autonomic Neuropathies (HSAN), among others. Collectively termed non-syndromic inherited peripheral neuropathies (NSIPN) in this article, these disorders represent the most common group of inherited neuromuscular diseases with an estimated prevalence of 1 in 2500 [1]. Over the past 30 years, a huge revolution in molecular genetics and genomics has occurred. Consequently, more than 100 NSIPN causing genes were identified with many different types of mutations and the number is still increasing [2,3]. In addition, several genes involved in one type of neuropathy were identified later in other phenotypes. For example, *SPTLC1*, reported for the first time as HSAN1A, was recently involved in the HMSN phenotype [4].

### 1.2. Molecular Diagnosis Is Positive around 40% of Cases

NSIPN represent a number of challenges for molecular diagnostic in laboratories, due to the clinical and genetic heterogeneity. To date, numerous studies worked on the rate of molecular diagnosis since the beginning of NGS analysis. In our previous study, we were able to make a molecular diagnosis in 40% (49/123 patients) with a gene panel list of 81 genes which were consistent with others studies [5]. Hartley et al. found 24% of diagnosis (12/50 families) by using whole exome sequencing (WES) [6]. Gonzagua-Jauregui et al. were able to have a positive rate in 46% (17/37 families) by WES [7]. Dorhn et al. used a targeted panel between 54–84 genes and identified the genetic cause in 19.8% (121/612 patients) [8]. By associating Multiplex-ligation-dependent-probe-amplification (MLPA) for *PMP22/GJB1/MPZ* and *GJB1/MPZ/PMP22,* Sanger sequencing, and targeted panel sequencing exclusively on CMT axonal, Padilha et al. found a molecular diagnosis in 55% of families (33/55) with a gene list panel of 104 genes [9]. Taghizadeh et al. were able to find a molecular diagnosis in 46.6% patients (27/58) by WES [10]. Cortese et al. found two diagnosis rates depending on regions, 32% in London with a panel of 50 genes and 30% in Iowa with a panel of 51 ± 23 genes [11]. 

The 60% undiagnosed cases might be caused by other types of mutations in known genes not identified, such as deep intronic variations and indels or by mutations in unknown genes. 

### 1.3. Next Generation Sequencing Technologies Increase the Rate of Molecular Diagnosis

Next generation sequencing (NGS) has revolutionized genomic research. Nowadays, NGS has been implemented and is largely used in day to day clinical practice. It provides possibilities for a more efficient genetic diagnostic service to patients with hereditary neuropathies [5]. Indeed, NGS platforms can perform parallel sequencing of millions of small DNA fragments. Furthermore, the NGS sequencing cost for large batches of samples is permanently decreasing and its efficiency growing regarding the time taken to sequence genetic material compared with the traditional Sanger sequencing method. To summarize, NGS method allows rapid and relatively cheap parallel sequencing of multiple genomic loci and the detection of a larger spectrum in DNA variations, providing unknown genetic variants, which is essential in the context of the genetic heterogeneity of CMT. 

In clinical CMT practices, customized targeted panels of disease-relevant gene is the most commonly used method for the NGS approach and offers a high degree of coverage of the selected genes [12]. However, because the mutational screening capacities in the field of neuropathies are exponentially increasing and the analysis of a gene list from a targeted panel associated with specific disease groups evolves rapidly. 

Due to the overlapping phenotypes of numerous genes in NSIPN, we choose the NGS analysis of a unique gene panel including genes involved in the three different subgroups.

## 2. Materials and Methods

Identical Gene List Panel Must Be Performed in Expert Laboratories 

In France, several genetic diagnostic laboratories are using NGS for targeted panels of disease-relevant genes in the NSIPN diagnosis. However, depending on their laboratory expertise and local history, these gene panels can be slightly different from one institute to another. 

FILNEMUS (Filière Nationale des Maladies Rares Neuromusculaires) was created in 2014 with the National Network for Rare Disease. One of its multiple missions has been to standardize the NGS diagnostic approach in order to standardize the testing procedure for patients from different regions. In this context, the nine French genetic diagnostic laboratories using NGS for myopathies recently published a consensual gene list [13]. Similarly, in this study, we aimed to establish a simple and accurate gene classification, more comprehensible and accessible for the whole clinical community, to provide a recommended specific “NSIPN-related disease gene list” to the reference diagnostic centres. We present an updated list of genes called “unique gene panel” based on the recommendation from seven specialized neuropathies diagnostic laboratories in France for NSIPN, including the three subgroups: HMSN, dHMN, and HSAN.

## 3. Results and Discussion

### 3.1. The FILNEMUS Consortium Implemented a Unique Gene Panel for NSIPN

The FILNEMUS consortium was a real springboard for the seven French neuropathy diagnostic laboratories using the NGS approach for NSIPN.

A concerted decision was achieved to identify a unique gene panel including genes involved in one or several of the three subgroups: (1) HMSN, (2) dHMN, and (3) HSAN. Each gene was selected through the Gene Table of Neuropathological Disorders [14], the practical experience from each of the seven diagnosis groups, and finally the available literature. Following these criteria, 127 genes were classified into NSIPN; 81 genes reported in HMSN, 26 in dHMN, and 20 in HSAN (Table 1) with overlap between the different subgroups. For example, *TRPV4* is reported in CMT and dHMN, *SCN9A* in HSAN and congenital insensitivity and more (see column “Diseases” in supplementary data Table S1, from references 1 to 281).

### 3.2. Molecular Strategy Is Based on Four Steps

**Figure 1:**
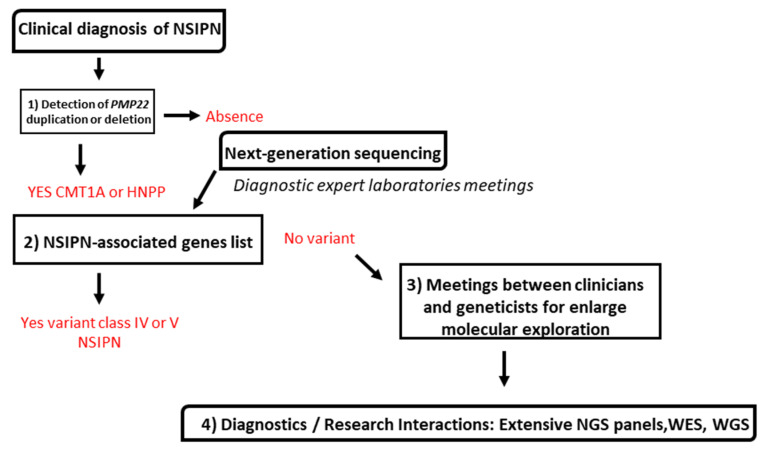
National French consensus strategy for genetic diagnosis of NSIPN using NGS.

For NSIPN, the standardized strategy consists in first (1) performing a rapid and reliable detection of duplication/deletion of the *PMP22* gene in patients with relevant phenotype as HMSN (Figure 1). 

In case of duplication, *PMP22* absence and depending on the prescription and clinical data, (2) an NGS analysis of a unique gene panel is performed.

Clinical data of NSIPN, family history, and conduction velocity of the median nerve are documented to help biologists interpret NGS data. NGS results generate a variants list classified according to American college of medical genetics ACMG criteria [15]. (3) Diagnostic pluridisciplinary meetings between clinicians and geneticists are organised to discuss the result and the pursuit or not of a molecular diagnosis for the patient. More precisely, in case we identify a class IV variant (likely pathogenic) or a class V variant (pathogenic) variant according to the ACMG classification, the analysis is stopped and we correlate the genotype with the phenotype of the patient. When a class III variant is identified (Variant of Unknown Significance), we discuss about the potentiality of this variant to be linked to the phenotype of the patient. If correlation occurs, we ask for familial study and functional experiments (if available). If no correlation takes place, we ask for additional investigations. In the absence of a potential variant or if the variant does not correspond to the phenotype, the meeting discussion turns toward the identification of new genes, (4) by performing WES or Whole Genome sequencing (WGS). 

### 3.3. Genes Are Classified Depending on Literature Evidence Based on Strande Publication and ClinGen Evaluation

**Figure 2:**
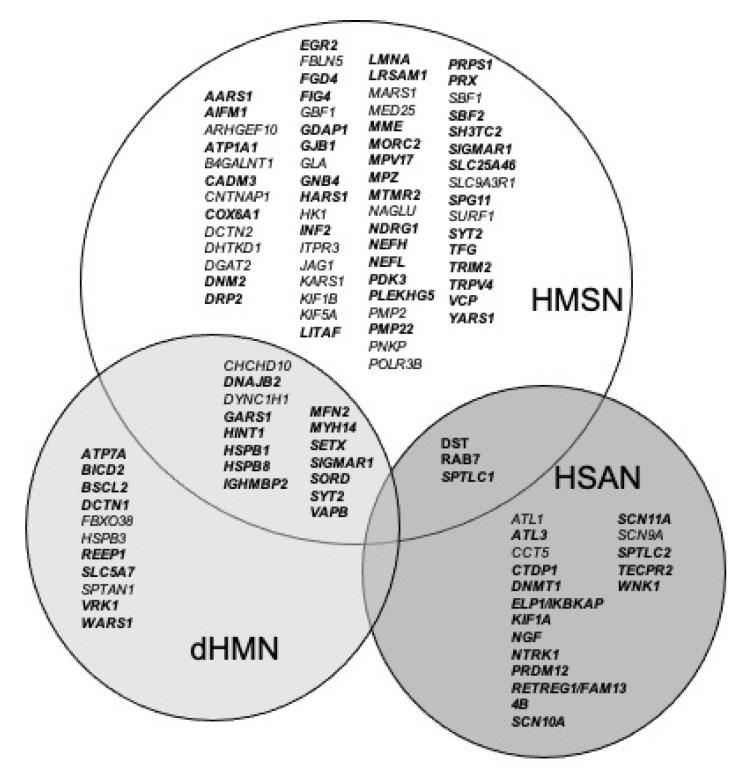
Venn Diagram of CMT gene subgroups. Bold genes represent the “definitive” classified genes.

We reported first the ClinGen evaluation (https://www.clinicalgenome.org/, accessed on 15 December 2021). ClinGen classified gene as definitive when a strict correlation is well established between the phenotype and the genotype, limited when not, and moderate when between both. For the genes not evaluated yet by ClinGen, we classified them as “definitive” if a minimum of two clinical studies were reported, associated with one report of functional evidence [16], such as, for example, for the *MPZ* (Myelin Protein Zero) gene. We chose papers reporting genes involving several families and we took into account the pathogenicity of the variants described with low or absence gnomAD (https://gnomad.broadinstitute.org/, accessed on 15 December 2021) occurrence. We classified genes as “limited” if there was not enough bibliographic data (one or two clinical cases without functional evidence/one clinical case and one functional evidence), such as, for example, the *PNKP* (Polynucleotide Kinase Phosphatase) gene. An important point is that some genes are involved in different phenotypes and can be “definitive” in one type of neuropathy and “limited” in another. For example, *DYNC1H1* is classified as “limited” in HMSN and “definitive” in dHMN. There is a broad clinical overlap in axonal HMSN and dHMN with some common genes involved as *DYNC1H1*, *GARS1*, *HSPB1*, *HSPB8*, *IGHMBP2*, *MFN2*, and *PLEKHG5,* as represented in Figure 2. This point led us to consider a unique gene panel for this disease’s group. We also observed a phenotypic overlap between NSIPN, but also with other diseases, such as distal myopathies or hereditary spastic paraplegia (HSP), as described in Pipis et al. [12,17]. A limitation of this classification is that some genes with strong clinical evidence are classified as “limited” due to poor functional evidence, such as *HK1*. Another important point to notice is that two genes *MED25* and *KIF1B* were disputed and no more involved in HMSN and “replaced” by *PNKP* and *MFN2* in their locus.

### 3.4. This Classification Allows Stratification of Variant Analysis

At least, 93 genes can be classified as “definitive” with, in detail: 55/81 genes in the HMSN group (68%), 21/26 in the dHMN group (81%), and 17/20 genes in the HSAN group (85%) leading to an overall 73% of genes having enough scientific evidence (Table 1). Compared to the myopathy consensus which defined 70% of definitive genes [13], we have the same literature proof for the molecular diagnosis of NSIPN. Through this classification, an accurate initial determination of the patient’s entry diagnosis allows the genetic screening of the most relevant genes potentially involved. This consensual diagnostic strategy was validated by the expert clinician group and geneticists and adopted by all participating laboratories.

## 4. Conclusions

### How Does This Consortium Helps Molecular Diagnosis in NSIPN?

This consensus is likely to increase the diagnostic rate and further our understanding of the genetic basis of NSIPN diseases as well. The burden of variable interpretation is considerable, and robust filtering strategies that use all the available clinical, genetic, and bioinformatics informations are required to classify variants.

Thanks to the nationwide FILNEMUS genetic diagnosis working-group, a yearly update of the consensual gene lists will be done. In this regard, the aim of this working group is to gradually classify all “gene-related diseases” associations according to the published ClinGen Clinical Validity framework. 

Within the next four years, we believe that the rapid expansion of NGS platforms across France with the implementation of bioinformatics tools to analyse the large volume of data generated will allow us to combine the WES and WGS strategy to the diagnosis of NSIPN, using this unique gene panel for an in silico first-step variant analysis. These techniques are very useful to increase the diagnostic yield in NSIPN. However, accurate and precise phenotyping of patients are mandatory for the NGS approach. Skipping this step leads to false diagnoses and increases diagnostic wavering.

This unique gene panel recommendation is thought to be a real improvement for the diagnosis. Our goal is to provide access to accurate diagnosis and appropriate treatment.

## Figures and Tables

**Table 1:** Summary of National French consensual gene lists for the genetic diagnosis of NSIPN.

Subgroup	Definitive Genes *n* (%)	Limited Genes *n* (%)	Total Genes *n*
CMT or HMSN	55 (68%)	26 (32%)	81
dHMN	21(81%)	5(19%)	26
HSAN	17(85%)	3(15%)	20
NSIPN	93 (73%)	34 (27%)	127

## Data Availability

The data are not available freely. Enquiries and requests for further information should be made to corresponding author.

## Supplementary Materials

The following supporting information can be downloaded at: https://www.mdpi.com/article/10.3390/genes13020318/s1, Table S1: National French consensual gene lists for the genetic diagnosis of NSIPN.

## Abbreviations

NGS	Next Generation Sequencing
CMT	Charcot–Marie–Tooth disease
HMSN	Hereditary Motor and Sensory Neuropathies
dHMN	Distal Hereditary Motor Neuropathies
HSAN	Hereditary Motor Neuropathies
DNA	Deoxyribonucleic Acid
FILNEMUS	Filière Nationale des Maladies Rares Neuromusculaires
PMP22	peripheral myelin protein 22
MPZ	Myelin Protein Zero
PNKP	Polynucleotide Kinase Phosphatase
WES	Whole Exome Sequencing
WGS	Whole Genome Sequencing
MLPA	Multiplex-ligation-dependent-probe-amplification
DYNC1H1	Dynein Cytoplasmic 1 Heavy Chain 1
GARS	Glycyl-TRNA Synthetase 1
HSPB	Heat Shock Protein Family
IGHMBP2	Immunoglobulin Mu DNA Binding Protein
MFN2	Mitofusin 2
PLEKHG5	Pleckstrin Homology And RhoGEF Domain Containing G5
SPG11	Spastic Paraplegia 11
VUS	Variant of Uncertain Significance
