# Supplementary material for: A National French Consensus on Gene List for the Diagnosis of Charcot–Marie–Tooth Disease and Related Disorders Using Next-Generation Sequencing
Source: Genes (Basel). 2022 Feb 9;13(2):318. doi: 10.3390/genes13020318 (PMC8871532; doi:10.3390/genes13020318)
Supplement: Supplementary file 1 [file genes-13-00318-s001.zip › genes-1565631-SI.pdf]

GENE SYMBOL	GENE NAME	RefSeq NM_DIAG	DISEASE*	INHERITANCE	ClinGen evaluation	Classification	Genetic evidence PMIDs/Notes 2 Publications	Experimental evidence PMIDs/ Note 1 publication
AARS1	Alanyl-tRNA-synthetase	NM_001605	CMT 2N	AD		Definitive	20045102[1] 22206013[2]	22009580 [3]
AIFM1	Apoptosis inducing factor mitochondria associated 1	NM_145812	CMT X4	X	Definitive	Definitive	20362274[4] 23217327[5]	20362274[4]
ARHGEF10	Rho guanine nucleotide exchange factor (GEF) 10	NM_014629	HMSN /Slow nerve conduction velocity	AD	Limited	Limited	14508709 [6]	21719701 [7]
ATL1	Atlastin GTPase 1	NM_001127713	HSAN 1D / SPG 3A	AD		Limited	21194679[8] 22340599[9]	33287888 [10]
ATL3	Atlastin GTPase 3	NM_015459	HSAN 1F	AD	Moderate	Definitive	24459106[11] 24736309[12]	30339187 [13]
ATP1A1	ATPase, Na+/K+ transporting, alpha 1 polypeptide	NM_000701	CMT2 DD	AD		Definitive	29499166 [14] 31373411 [15]	31707753 [16]
ATP7A	ATPase, Cu++ transporting, alpha polypeptide	NM_000052	dHMN / SMA	XLD		Definitive	20170900[17] 31558336[18]	31969342 [19]
B4GALNT1	Beta-1,4-N-acetyl-galactosaminyltransferase 1	NM_001478	HSP 26 / CMT2	AR		Limited	28536081[20] 34595861[21]	
BICD2	Bicaudal D homolog 2 (Drosophila)	NM_001003800	SMALED 2A / 2B dHMN	AD		Definitive	23664116[22] 23664119[23] 30536747[24]	27784775 [25]
BSCL2	Berardinelli-Seip congenital lipodystrophy 2 (seipin)	NM_001122955	dHMN 5A	AD	Definitive	Definitive	14981520[26] 26815532[27]	17387721 [28]

CADM3	Cell adhesion molecule 3	NM_001127173	CMT 2FF	AD		Limited	33889941[29]	
CCT5	Chaperonin containing TCP1, subunit 5 (epsilon)	NM_012073	HSAN SP	AR		Limited	16399879[30]	25345891 [31]
CHCHD10	Coiled-coil-helix-coiled-coil-helix domain containing 10	NM_213720	SMA Jokela/ CMT 2	AD		Limited	25428574[32] 27066538[33]	
CNTNAP1	contactin associated protein 1	NM_003632	CMT 2/ congenital hypomyelinating neuropathy type 3 (CHN3)	AR		Limited	28374019[34] 31397905[35]	
COX6A1	Cytochrome c oxidase subunit VIa polypeptide 1	NM_004373	CMT ID	AR		Definitive	25152455 [36] 26302975 [37]	25152455 [36]
CTDP1	CTD (carboxy-terminal domain, RNA polymerase II, polypeptide A) phosphatase, subunit 1	NM_004715	CCFDN	AR		Definitive	14517542 [38] 24690360 [39]	14517542 [38]
DCTN1	Dynactin 1	NM_004082	dHMN VIIB	AD		Definitive	12627231 [40] 27573046 [41]	16505168 [42]
DCTN2	Dynactin 2	NM_001261413	CMT I	AD		Limited	26517670[43]	
DHTKD1	Dehydrogenase E1 and transketolase domain containing 1	NM_018706	CMT 2Q	AD		Limited	23141294 [44]	29661920 [45]
DGAT2	diacylglycerol O-acyltransferase 2	NM_032564	CMT 2	AD		Limited	31372974[46]	26786738[47]
DNAJB2	DnaJ (Hsp40) homolog, subfamily B, member 2	NM_006736	SMA 5	AR		Definitive	22522442 [48] 25274842 [49]	22522442 [48]

DNM2	Dynamin 2	NM_001005361	CMT 2N / CMT IB / Lethal congenital contracture syndrome	AD ~~AR~~	Definitive	Definitive	15731758 [50] 17636067 [51]	26842864 [52]
DNMT1	DNA (cytosine-5-)-methyltransferase 1	NM_001130823	HSAN 1E	AD		Definitive	21532572 [53] 23365052 [54]	21532572 [53]
DRP2	dystrophin related protein 2	NM_001939	CMT I	X		Definitive	26227883[55] 31217940[56]	29623298[57]
DST	Dystonin	NM_001144769	HSAN 6	AR	Definitive	Definitive	22522446 [58] 32802955[59]	29982604 [60]
DST	Dystonin	NM_001144769	HMSN	AR		Limited	32802955[59]	
DYNC1H1	Dynein, cytoplasmic 1, heavy chain 1	NM_001376	CMT 2O	AD		Limited	21820100 [61]	
DYNC1H1	Dynein, cytoplasmic 1, heavy chain 1	NM_001376	HMSN	AD		Definitive	22459677 [62] 22847149 [63]	23742762 [64]
EGR2	Early growth response 2	NM_000399	CMT 1D / Dejerine-Sottas Disease / Hypomyelinating congenital neuropathy 1	AD / AD;AR / AD;AR	Definitive	Definitive	9537424 [65] 10371530 [66]	7935840 [67]
ELP1/ IKBKAP	Inhibitor of kappa light polypeptide gene enhancer in B-cells, kinase complex-associated protein	NM_003640	FD	AR		Definitive	11179008 [68] 12687659 [69]	17644305[70]
FBLN5	Fibulin 5	NM_006329	HNARMD / CMT1	AD		Limited	21576112 [71] 23328402 [72] 28332470 [73]	33107705 [74]
FBXO38	F-box protein 38	NM_030793	SMALED 1 / dHMN2D	AD/AR		Limited	24207122 [75] 31420593 [76]	
FGD4	FYVE, RhoGEF and PH domain containing 4	NM_139241	CMT 4H	AR	Definitive	Definitive	15744041 [77] 17564972 [78]	17564959 [79]

FIG4	Phosphoinositide 5-phosphatase	NM_014845	CMT 4J	AR	Definitive	Definitive	17572665 [80]<br>21705420 [81]	18556664 [82]
GARS1	Glycyl-tRNA synthetase	NM_002047	CMT 2D /<br>dSMA V	AD /<br>AD		Definitive	8872480 [83]<br>12690580 [84]	25168514 [85]<br>26503042 [86]
GBF1	Golgi brefeldin A resistant guanine nucleotide exchange factor 1	NM_001377137	CMT2GG	AD		Limited	32937143[87]	32937143[87]
GDAP1	Ganglioside induced differentiation associated protein 1	NM_018972	CMT 2K /<br>CMT 4A	AD/<br>AR	Definitive	Definitive	11743579 [88]<br>15805163 [89]	21753178 [90]
GJB1	Gap junction protein, beta 1, 32kDa	NM_001097642	CMT X	X	Definitive	Definitive	8266101 [91]<br>10732813 [92]	15852376 [93]
GLA	Galactosidase alpha	NM_000169	Fabry disease		Definitive	Definitive	27164712[94]<br>12359124[95]	12359124[95]
GNB4	Guanine nucleotide binding protein (G protein), beta polypeptide 4	NM_021629	CMT IF	AD	Moderate	Definitive	23434117 [96]<br>27908631 [97]	23434117 [96]
HARS1	Histidyl-tRNA synthetase	NM_002109	CMT 2W	AD		Definitive	22930593 [98]<br>26072516 [99]	22930593 [98]
HINT1	Histidine triad nucleotide binding protein 1	NM_005340	ARAN-NM	AR	Definitive	Definitive	22961002 [100]<br>25342199 [101]	22961002 [100]
HK1	Hexokinase 1	NM_033497	HMSN type Russe	AR		Limited	19536174 [102]<br>22978647 [103]	
HSPB1	Heat shock 27kDa protein 1	NM_001540	CMT 2F/dHMN IIB	AD		Definitive	15122254 [104]<br>18832141 [105]	21785432 [106]
HSPB3	Heat shock 27kDa protein 3	NM_006308	HMN 2C	AD		Limited	20142617 [107]<br>29341343 [108]	27567740 [109]

HSPB8	Heat shock 22kDa protein 8	NM_014365	CMT 2L	AD		Definitive	15122253 [110] 15565283 [111]	20538880 [112]
HSPB8	Heat shock 22kDa protein 8	NM_014365	dHMN IIA	AD	Definitive	Definitive	15122253 [110] 15565283 [111]	20538880 [112]
IGHMBP2	Immunoglobulin mu binding protein 2	NM_002180	CMT 2S	AR		Definitive	25439726 [113] 25568292 [114]	25439726 [113]
IGHMBP2	Immunoglobulin mu binding protein 2	NM_002180	dHMN VI	AR		Definitive	25439726 [113] 25568292 [114]	25439726 [113]
INF2	Inverted formin, FH2 and WH2 domain containing	NM_022489	CMT IE	AD	Definitive	Definitive	22187985 [115] 30680856 [116]	22187985 [115]
ITPR3	inositol 1,4,5-trisphosphate receptor type 3	NM_002224	CMT I	AD		Limited	32949214[117]	
JAG1	canonical Notch ligand Jagged1	NM_000214	CMT 2H	AD		Limited	32065591[118]	
~~*JPH1*~~	~~Junctophilin 1~~	~~NM_020647~~	~~CMT 2K~~	~~AD~~		~~Definitive~~		
KARS1	Lysyl-tRNA synthetase	NM_001130089	CMT IB	AR		Limited	20920668 [119]	
KIF1A	Kinesin family member 1A	NM_001244008	HSAN 2C / HSP 30	AR		Definitive	21820098 [120] 22258533 [121]	27263974 [122]
KIF1B	Kinesin family member 1B	NM_015074	CMT 2A1	AD	No Known Disease Relationship	Limited	11389829 [123] 25802885 [124]	19503091 [125] 30126838 [126]
KIF5A	Kinesin family member 5A	NM_004984	CMT 2/ HSP 10	AD		Limited	25008398[127] 29892902[128]	
LITAF	Lipopolysaccharide-induced TNF factor	NM_001136472	CMT 1C	AD	Moderate	Definitive	12525712 [129] 15776429 [130]	23359569 [131]
LMNA	Lamin A/C	NM_170707	CMT 2B1	AR		Definitive	11799477 [132] 17536044 [133]	11799477 [132]
LRSAM1	Leucine rich repeat and sterile alpha motif containing 1	NM_001005374	CMT 2P	AD / AR		Definitive	20865121 [134] 27686364 [135]	22012984 [136]

MARS1	Methionyl-tRNA synthetase	NM_004990	CMT 2U	AD	Limited	Limited	23729695 [137] 24354524 [138]	31356216 [139]
MED25	Mediator complex subunit 25	NM_030973	CMT2B2	AR	Disputed	Disputed	19290556[140] 30039206[141]	
MFN2	Mitofusin 2	NM_001127660	CMT 2A2A / CMT 2A2B	AD / AR		Definitive	15064763 [142] 16835246 [143]	17959936 [144]
MFN2	Mitofusin 2	NM_001127660	dHMN VIA	AD / AR		Definitive	15064763 [142] 16835246 [143]	17959936 [144]
MME	Membrane metallo-endopeptidase	NM_007288	CMT 2T	AD AR		Definitive in AR inheritance / Limited in AD inheritance	26991897 [145] 27588448 [146]	27588448 [146]
MORC2	MORC family CW-type zinc finger 2	NM_014941	CMT 2Z	AD		Definitive	26497905 [147] 26659848 [148]	30624633 [149]
MPV17	Mitochondrial inner membrane protein 17	NM_002437	CMT 2	AR		Definitive	26437932[150] 30298599[151]	26437932[150]
MPZ	Myelin protein zero	NM_000530	CMT 1B CMT 2I	AD	Definitive	Definitive	7693129 [152] 19293842 [153]	10704451 [154]
MTMR2	Myotubularin related protein 2	NM_016156	CMT 4B1	AR	Definitive	Definitive	10802647 [155] 28509084 [156]	15557122 [157]
MYH14	Myosin, heavy chain 14, non-muscle	NM_001145809	PNMHH	AD		Definitive	21480433 [158] 27875632 [159]	31231018 [160]
NAGLU	N-acetylglucosaminidase, alpha	NM_000263	CMT 2V	AD		Limited	25818867 [161]	25818867 [161]
NDRG1	N-myc downstream regulated 1	NM_006096	CMT 4D	AR		Definitive	10831399 [90] 12872253 [163]	15082788 [164]
NEFH	Neurofilament, heavy polypeptide	NM_021076	CMT 2CC	AD	Definitive	Definitive	27040688 [165] 30992180 [166]	27040688 [165]
NEFL	Neurofilament, light polypeptide	NM_006158	CMT 2E / CMT 1F / CMT IG	AD / AD-AR		Definitive	10841809 [167] 12566280 [168]	12393795 [169]
NGF	Nerve growth factor (beta polypeptide)	NM_002506	HSAN 5	AR		Definitive	14976160 [170] 20978020 [171]	29483280 [172]

NRG2	Neuregulin 2	NM_004883		AR		No correlation with CMT disease	10369162[173]	
NTRK1	Neurotrophic tyrosine kinase, receptor, type 1	NM_002529	CIPA / HSAN 4	AR	Definitive	Definitive	10330344 [174]<br>28177573 [175]	27676246 [176]
PDK3	Pyruvate dehydrogenase kinase, isozyme 3	NM_001142386	CMT X6	AD		Definitive	23297365 [177]<br>26801680 [178]	23297365[177]
PHYH	Phytanoyl-coa hydroxylase	NM_006214	Refsum disease	AR		Definitive	11555634[179]<br>32773395[180]	
PLEKHG5	Pleckstrin homology domain containing, family G (with RhoGef domain) member 5	NM_001265593	CMT IC	AR		Definitive	23777631 [181]<br>23844677 [182]	
PMP2	Peripheral myelin protein 2	NM_002677	CMT 1G	AD	Limited	Limited	26257172 [183]<br>26828946 [184]	26828946 [184]
PMP22	Peripheral myelin protein 22	NM_153322	CMT 1A /<br>CMT 1E /<br>DSS /<br>HNPP	AD /<br>AR in some DSS		Definitive	(Chicago:Mosby-Yearbook) [185]<br>1303230 [186]<br>**32538861** [187]	1999826 [187]<br>32538861[188]
PNKP	Polynucleotide kinase 3'-phosphatase	NM_007254	CMT 2B2	AR		Limited	23224214[189]<br>30039206[141]	
POLR3B	RNA polymerase 3 subunit B	NM_018082	CMT 1	AD		Limited	33417887[190]<br>34666706[191]	
PRDM12	PR domain containing 12	NM_021619	HSAN 8	AR		Definitive	26005867 [192]<br>26975306 [193]	25891934 [194]
PRPS1	Phosphoribosyl pyrophosphate synthetase 1	NM_002764	CMT X5	X		Definitive	17701900 [195]<br>20380929 [196]	31434166 [197]
PRX	Periaxin	NM_181882	CMT 4F /<br>DSS	AR /<br>AD-AR		Definitive	10848494 [198]<br>15197604 [199]	10839370 [200]
RAB7A	RAB7A, member RAS oncogene family	NM_004637	CMT 2B	AD		Definitive	12545426 [201]<br>15455439 [202]	24521780 [203]

REEP1	Receptor accessory protein 1	NM_022912	HMN 5B / SPG31	AD / AD		Definitive	22703882 [204] 30637453 [205]	22703882 [204]
RETREG1/ FAM134B	Family with sequence similarity 134, member B	NM_001034850	HSAN 2B	AR		Definitive	19838196 [206] 24327336 [207]	26040720 [208]
SBF1	SET binding factor 1	NM_002972	CMT 4B3	AR	Moderate	Limited	23749797 [209] 24799518 [210]	
SBF2	SET binding factor 2	NM_030962	CMT 4B2	AR	Definitive	Definitive	12554688 [211] 12687498 [212]	17855448 [213]
SCN10A	Sodium channel, voltage-gated, type X, alpha subunit	NM_006514	FEPS 2	AD		Definitive	23115331 [214] 29448912 [215]	23115331 [214]
SCN11A	Sodium channel, voltage-gated, type XI, alpha subunit	NM_014139	HSAN 7	AD		Definitive	24036948 [216] 25118027 [217]	15964986 [218]
SCN9A	Sodium channel, voltage-gated, type IX, alpha subunit	NM_002977	HSAN 2D / CIPA Small fiber neuropathy (SFN)	AR AD in SFN		Limited	20692858 [219] 23596073 [220]	
SETX	Senataxin	NM_015046	SCAN2	AR		Definitive	15732101 [221] 14770181 [222]	19593598 [223]
SH3TC2	SH3 domain and tetratricopeptide repeats 2	NM_024577	CMT 4C	AR		Definitive	14574644 [224] 16326826 [225]	19805030 [226]
SIGMAR1	Sigma non-opioid intracellular receptor 1	NM_005866	SMA 2	AR		Definitive	26078401 [227] 28503617 [228]	27402882 [229]
SLC25A46	Solute carrier family 25 member 46	NM_138773.4	HSMN6B	AR	Definitive	Definitive	26168012[230] 27430653[231]	26168012[230]
SLC5A7	Solute carrier family 5 (sodium/choline cotransporter), member 7	NM_021815	HMN 7A	AD	Moderate	Definitive	23141292 [232] 29782645 [233]	23141292 [232]
SLC9A3R1	SLC9A3 regulator 1	NM_004252	CMT1			Limited	32077526[234]	
SOD1	Superoxide dismutase 1	NM_000454.5	HMSN			Limited	9462467[235]	

SORD	Sorbitol dehydrogenase	NM_003104	dHMN/ CMT 2	AR		Definitive	32367058 [236]<br>33397963 [237]	32367058 [236]
SPG11	Spastic paraplegia 11	NM_025137	CMT 2X	AR		Definitive	26556829 [238]<br>27544499 [239]	28237315 [240]
SPG11	Spastic paraplegia 11	NM_025137	SPG	AR		Definitive	14732628[241]	28237315 [240]
SPTAN1	Alpha II spectrin	NM_001130438	HMN	AD		Limited	31332438[242]<br>33206935[243]	
SPTLC1	Serine palmitoyltransferase, long chain base subunit 1	NM_006415	HSAN 1A	AD		Definitive	11242114 [244]<br>11242106 [245]	16210380 [246]
SPTLC2	Serine palmitoyltransferase, long chain base subunit 2	NM_004863	HSAN 1C	AD		Definitive	20920666 [247]<br>23658386 [248]	20920666 [247]
SURF1	Surfeit 1	NM_003172	CMT 4K	AR		Limited	24027061 [249]	
SYT2	Synaptotagmin II	NM_177402	CMS 7	AD		Definitive	26519543 [250]<br>30533528 [251]	30533528 [251]
TECPR2	Tectonin β-propeller repeat-containing protein	NM_014844	HSAN 3	AR		Definitive	26542466[252]<br>33847017[253]	33218264[254]
TFG	TRK-fused gene	NM_001195478	HMSN Okinawa	AD		Definitive	22883144 [255]<br>23553329 [256]	25098539 [257]
TRIM2	Tripartite motif containing 2	NM_015271	CMT 2R	AR		Definitive	23562820 [258]<br>25893792 [259]	18687884 [260]

TRPV4	Transient receptor potential cation channel, subfamily V, member 4	NM_021625	CMT 2C	AD	Definitive	Definitive	20037588 [261]<br>20037587 [262]	21288981 [263]
TTR	Transthyretin	NM-000371	Amyloidosis hereditary	AD	Definitive	Definitive	11385707[264]<br>1848299[265]	15820680[266]
VAPB	VAMP (vesicle-associated membrane protein)-associated protein B and C	NM_004738	ALS 8 / SMA Finkel Type	AD		Definitive	15372378 [267]<br>20940299 [268]	26362257 [269]
VCP	Valosin containing protein	NM_007126	CMT 2Y	AD		Definitive	25125609 [270]<br>25878907 [271]	25125609 [270]
VRK1	Vaccinia related kinase 1	NM_003384	SMA	AR		Definitive	24126608 [272]<br>30847374 [273]	31090908[274]
VRK1	Vaccinia related kinase 1	NM_003384	dHMN	AR		Definitive	31090908[274]<br>34990802[275]	31090908 [274]
WARS1	Tryptophanyl-tRNA synthetase	NM_173701	dHMN IX	AD		Definitive	28369220 [276]<br>31069783 [277]	28369220 [276]
WNK1	WNK lysine deficient protein kinase 1	NM_001184985	HSAN 2	AR		Definitive	15060842 [278]<br>15911806 [279]	23300475 [280]
YARS1	Tyrosyl-tRNA synthetase	NM_003680	CMT IC	AD	Definitive	Definitive	16429158 [281]<br>24354524 [138]	19561293 [282]

Supplementary Table S1: National French consensual gene list for the genetic diagnosis of NSIPN

References:

1. Latour, P.; Thauvin-Robinet, C.; Baudelet-Méry, C.; Soichot, P.; Cusin, V.; Faivre, L.; Locatelli, M.-C.; Mayençon, M.; Sarcey, A.; Broussolle, E.; et al. A Major Determinant for Binding and Aminoacylation of TRNA(Ala) in Cytoplasmic Alanyl-TRNA Synthetase Is Mutated in Dominant Axonal Charcot-Marie-Tooth Disease. *Am. J. Hum. Genet.* **2010**, *86*, 77–82, doi:10.1016/j.ajhg.2009.12.005.
2. Lin, K.-P.; Soong, B.-W.; Yang, C.-C.; Huang, L.-W.; Chang, M.-H.; Lee, I.-H.; Antonellis, A.; Antonellis, A.; Lee, Y.-C. The Mutational Spectrum in a Cohort of Charcot-Marie-Tooth Disease Type 2 among the Han Chinese in Taiwan. *PLoS ONE* **2011**, *6*, e29393, doi:10.1371/journal.pone.0029393.
3. McLaughlin, H.M.; Sakaguchi, R.; Giblin, W.; NISC Comparative Sequencing Program; Wilson, T.E.; Biesecker, L.; Lupski, J.R.; Talbot, K.; Vance, J.M.; Züchner, S.; et al. A Recurrent Loss-of-Function Alanyl-TRNA Synthetase (AARS) Mutation in Patients with Charcot-Marie-Tooth Disease Type 2N (CMT2N). *Hum. Mutat.* **2012**, *33*, 244–253, doi:10.1002/humu.21635.
4. Ghezzi, D.; Sevrioukova, I.; Invernizzi, F.; Lamperti, C.; Mora, M.; D'Adamo, P.; Novara, F.; Zuffardi, O.; Uziel, G.; Zeviani, M. Severe X-Linked Mitochondrial Encephalomyopathy Associated with a Mutation in Apoptosis-Inducing Factor. *Am J Hum Genet* **2010**, *86*, 639–649, doi:10.1016/j.ajhg.2010.03.002.
5. Rinaldi, C.; Grunseich, C.; Sevrioukova, I.F.; Schindler, A.; Horkayne-Szakaly, I.; Lamperti, C.; Landouré, G.; Kennerson, M.L.; Burnett, B.G.; Bönnemann, C.; et al. Cowchock Syndrome Is Associated with a Mutation in Apoptosis-Inducing Factor. *Am J Hum Genet* **2012**, *91*, 1095–1102, doi:10.1016/j.ajhg.2012.10.008.
6. Verhoeven, K.; De Jonghe, P.; Van de Putte, T.; Nelis, E.; Zwijsen, A.; Verpoorten, N.; De Vriendt, E.; Jacobs, A.; Van Gerwen, V.; Francis, A.; et al. Slowed Conduction and Thin Myelination of Peripheral Nerves Associated with Mutant Rho Guanine-Nucleotide Exchange Factor 10. *Am. J. Hum. Genet.* **2003**, *73*, 926–932, doi:10.1086/378159.
7. Chaya, T.; Shibata, S.; Tokuhara, Y.; Yamaguchi, W.; Matsumoto, H.; Kawahara, I.; Kogo, M.; Ohoka, Y.; Inagaki, S. Identification of a Negative Regulatory Region for the Exchange Activity and Characterization of T332I Mutant of Rho Guanine Nucleotide Exchange Factor 10 (ARHGEF10). *J. Biol. Chem.* **2011**, *286*, 29511–29520, doi:10.1074/jbc.M111.236810.
8. Guelly, C.; Zhu, P.-P.; Leonardis, L.; Papić, L.; Zidar, J.; Schabhüttl, M.; Strohmaier, H.; Weis, J.; Strom, T.M.; Baets, J.; et al. Targeted High-Throughput Sequencing Identifies Mutations in Atlastin-1 as a Cause of Hereditary Sensory Neuropathy Type I. *Am J Hum Genet* **2011**, *88*, 99–105, doi:10.1016/j.ajhg.2010.12.003.
9. Leonardis, L.; Auer-Grumbach, M.; Papić, L.; Zidar, J. The N355K Atlastin 1 Mutation Is Associated with Hereditary Sensory Neuropathy and Pyramidal Tract Features. *Eur. J. Neurol.* **2012**, *19*, 992–998, doi:10.1111/j.1468-1331.2012.03665.x.
10. Mou, Y.; Dong, Y.; Chen, Z.; Denton, K.R.; Duff, M.O.; Blackstone, C.; Zhang, S.-C.; Li, X.-J. Impaired Lipid Metabolism in Astrocytes Underlies Degeneration of Cortical Projection Neurons in Hereditary Spastic Paraplegia. *Acta Neuropathol Commun* **2020**, *8*, 214, doi:10.1186/s40478-020-01088-0.
11. Kornak, U.; Mademan, I.; Schinke, M.; Voigt, M.; Krawitz, P.; Hecht, J.; Barvencik, F.; Schinke, T.; Gießelmann, S.; Beil, F.T.; et al. Sensory Neuropathy with Bone Destruction Due to a Mutation in the Membrane-Shaping Atlastin GTPase 3. *Brain* **2014**, *137*, 683–692, doi:10.1093/brain/awt357.
12. Fischer, D.; Schabhüttl, M.; Wieland, T.; Windhager, R.; Strom, T.M.; Auer-Grumbach, M. A Novel Missense Mutation Confirms ATL3 as a Gene for Hereditary Sensory Neuropathy Type 1. *Brain* **2014**, *137*, e286, doi:10.1093/brain/awu091.
13. Krols, M.; Asselbergh, B.; De Rycke, R.; De Winter, V.; Seyer, A.; Müller, F.-J.; Kurth, I.; Bultynck, G.; Timmerman, V.; Janssens, S. Sensory Neuropathy-Causing Mutations in ATL3 Affect ER-Mitochondria Contact Sites and Impair Axonal Mitochondrial Distribution. *Hum. Mol. Genet.* **2019**, *28*, 615–627, doi:10.1093/hmg/ddy352.

14. Lassuthova, P.; Rebelo, A.P.; Ravenscroft, G.; Lamont, P.J.; Davis, M.R.; Manganelli, F.; Feely, S.M.; Bacon, C.; Brožková, D.Š.; Haberlova, J.; et al. Mutations in ATP1A1 Cause Dominant Charcot-Marie-Tooth Type 2. *Am. J. Hum. Genet.* **2018**, *102*, 505–514, doi:10.1016/j.ajhg.2018.01.023.
15. He, J.; Guo, L.; Lin, S.; Chen, W.; Xu, G.; Cai, B.; Xu, L.; Hong, J.; Qiu, L.; Wang, N.; et al. ATP1A1 Mutations Cause Intermediate Charcot-Marie-Tooth Disease. *Hum. Mutat.* **2019**, *40*, 2334–2343, doi:10.1002/humu.23886.
16. Manganelli, F.; Parisi, S.; Nolano, M.; Miceli, F.; Tozza, S.; Pisciotta, C.; Iodice, R.; Provitera, V.; Cicatiello, R.; Zuchner, S.; et al. Insights into the Pathogenesis of ATP1A1-Related CMT Disease Using Patient-Specific IPSCs. *J. Peripher. Nerv. Syst.* **2019**, *24*, 330–339, doi:10.1111/jns.12357.
17. Kennerson, M.L.; Nicholson, G.A.; Kaler, S.G.; Kowalski, B.; Mercer, J.F.B.; Tang, J.; Llanos, R.M.; Chu, S.; Takata, R.I.; Speck-Martins, C.E.; et al. Missense Mutations in the Copper Transporter Gene ATP7A Cause X-Linked Distal Hereditary Motor Neuropathy. *Am. J. Hum. Genet.* **2010**, *86*, 343–352, doi:10.1016/j.ajhg.2010.01.027.
18. Gualandi, F.; Sette, E.; Fortunato, F.; Bigoni, S.; De Grandis, D.; Scotton, C.; Selvatici, R.; Neri, M.; Incensi, A.; Liguori, R.; et al. Report of a Novel ATP7A Mutation Causing Distal Motor Neuropathy. *Neuromuscul. Disord.* **2019**, *29*, 776–785, doi:10.1016/j.nmd.2019.08.008.
19. Perez-Siles, G.; Cutrupi, A.; Ellis, M.; Kuriakose, J.; La Fontaine, S.; Mao, D.; Uesugi, M.; Takata, R.I.; Speck-Martins, C.E.; Nicholson, G.; et al. Modelling the Pathogenesis of X-Linked Distal Hereditary Motor Neuropathy Using Patient-Derived IPSCs. *Dis Model Mech* **2020**, *13*, doi:10.1242/dmm.041541.
20. Dad, R.; Malik, U.; Javed, A.; Minassian, B.A.; Hassan, M.J. Structural Annotation of Beta-1,4-N-Acetyl Galactosaminyltransferase 1 (B4GALNT1) Causing Hereditary Spastic Paraplegia 26. *Gene* **2017**, *626*, 258–263, doi:10.1016/j.gene.2017.05.041.
21. Hong, J.M.; Jeon, H.; Choi, Y.C.; Cho, H.; Hong, Y.B.; Park, H.J. A Compound Heterozygous Pathogenic Variant in B4GALNT1 Is Associated With Axonal Charcot-Marie-Tooth Disease. *J Clin Neurol* **2021**, *17*, 534–540, doi:10.3988/jcn.2021.17.4.534.
22. Neveling, K.; Martinez-Carrera, L.A.; Hölker, I.; Heister, A.; Verrips, A.; Hosseini-Barkooie, S.M.; Gilissen, C.; Vermeer, S.; Pennings, M.; Meijer, R.; et al. Mutations in BICD2, Which Encodes a Golgin and Important Motor Adaptor, Cause Congenital Autosomal-Dominant Spinal Muscular Atrophy. *Am. J. Hum. Genet.* **2013**, *92*, 946–954, doi:10.1016/j.ajhg.2013.04.011.
23. Peeters, K.; Litvinenko, I.; Asselbergh, B.; Almeida-Souza, L.; Chamova, T.; Geuens, T.; Ydens, E.; Zimoń, M.; Irobi, J.; De Vriendt, E.; et al. Molecular Defects in the Motor Adaptor BICD2 Cause Proximal Spinal Muscular Atrophy with Autosomal-Dominant Inheritance. *Am. J. Hum. Genet.* **2013**, *92*, 955–964, doi:10.1016/j.ajhg.2013.04.013.
24. Kropatsch, R.; Schmidt, H.M.; Buttkereit, P.; Epplen, J.T.; Hoffjan, S. BICD2 Mutational Analysis in Hereditary Spastic Paraplegia and Hereditary Motor and Sensory Neuropathy. *Muscle Nerve* **2019**, *59*, 484–486, doi:10.1002/mus.26394.
25. Unger, A.; Dekomien, G.; Güttsches, A.; Dreps, T.; Kley, R.; Tegenthoff, M.; Ferbert, A.; Weis, J.; Heyer, C.; Linke, W.A.; et al. Expanding the Phenotype of BICD2 Mutations toward Skeletal Muscle Involvement. *Neurology* **2016**, *87*, 2235–2243, doi:10.1212/WNL.0000000000003360.
26. Windpassinger, C.; Auer-Grumbach, M.; Irobi, J.; Patel, H.; Petek, E.; Hörl, G.; Malli, R.; Reed, J.A.; Dierick, I.; Verpoorten, N.; et al. Heterozygous Missense Mutations in BSCL2 Are Associated with Distal Hereditary Motor Neuropathy and Silver Syndrome. *Nat. Genet.* **2004**, *36*, 271–276, doi:10.1038/ng1313.
27. Hsiao, C.-T.; Tsai, P.-C.; Lin, C.-C.; Liu, Y.-T.; Huang, Y.-H.; Liao, Y.-C.; Huang, H.-W.; Lin, K.-P.; Soong, B.-W.; Lee, Y.-C. Clinical and Molecular Characterization of BSCL2 Mutations in a Taiwanese Cohort with Hereditary Neuropathy. *PLoS ONE* **2016**, *11*, e0147677, doi:10.1371/journal.pone.0147677.
28. Ito, D.; Suzuki, N. Molecular Pathogenesis of Seipin/BSCL2-Related Motor Neuron Diseases. *Ann. Neurol.* **2007**, *61*, 237–250, doi:10.1002/ana.21070.
29. Rebelo, A.P.; Cortese, A.; Abraham, A.; Eshed-Eisenbach, Y.; Shner, G.; Vainshtein, A.; Buglo, E.; Camarena, V.; Gaidosh, G.; Shiekhattar, R.; et al. A CADM3 Variant Causes Charcot-Marie-Tooth Disease with Marked Upper Limb Involvement. *Brain* **2021**, *144*, 1197–1213, doi:10.1093/brain/awab019.

30. Bouhouche, A.; Benomar, A.; Bouslam, N.; Chkili, T.; Yahyaoui, M. Mutation in the Epsilon Subunit of the Cytosolic Chaperonin-Containing t-Complex Peptide-1 (Cct5) Gene Causes Autosomal Recessive Mutilating Sensory Neuropathy with Spastic Paraplegia. *J. Med. Genet.* **2006**, *43*, 441–443, doi:10.1136/jmg.2005.039230.
31. Min, W.; Angileri, F.; Luo, H.; Lauria, A.; Shanmugasundaram, M.; Almerico, A.M.; Cappello, F.; de Macario, E.C.; Lednev, I.K.; Macario, A.J.L.; et al. A Human CCT5 Gene Mutation Causing Distal Neuropathy Impairs Hexadecamer Assembly in an Archaeal Model. *Sci Rep* **2014**, *4*, 6688, doi:10.1038/srep06688.
32. Penttilä, S.; Jokela, M.; Bouquin, H.; Saukkonen, A.M.; Toivanen, J.; Udd, B. Late Onset Spinal Motor Neuronopathy Is Caused by Mutation in CHCHD10. *Ann. Neurol.* **2015**, *77*, 163–172, doi:10.1002/ana.24319.
33. Auranen, M.; Ylikallio, E.; Shcherbii, M.; Paetau, A.; Kiuru-Enari, S.; Toppila, J.P.; Tyynismaa, H. CHCHD10 Variant p.(Gly66Val) Causes Axonal Charcot-Marie-Tooth Disease. *Neurol Genet* **2015**, *1*, e1, doi:10.1212/NXG.0000000000000003.
34. Hengel, H.; Magee, A.; Mahanjah, M.; Vallat, J.-M.; Ouvrier, R.; Abu-Rashid, M.; Mahamid, J.; Schüle, R.; Schulze, M.; Krägeloh-Mann, I.; et al. CNTNAP1 Mutations Cause CNS Hypomyelination and Neuropathy with or without Arthrogryposis. *Neurol Genet* **2017**, *3*, e144, doi:10.1212/NXG.0000000000000144.
35. Freed, A.S.; Weiss, M.D.; Malouf, E.A.; Hisama, F.M. CNTNAP1 Mutations in an Adult with Charcot Marie Tooth Disease. *Muscle Nerve* **2019**, *60*, E28–E30, doi:10.1002/mus.26658.
36. Tamiya, G.; Makino, S.; Hayashi, M.; Abe, A.; Numakura, C.; Ueki, M.; Tanaka, A.; Ito, C.; Toshimori, K.; Ogawa, N.; et al. A Mutation of COX6A1 Causes a Recessive Axonal or Mixed Form of Charcot-Marie-Tooth Disease. *Am. J. Hum. Genet.* **2014**, *95*, 294–300, doi:10.1016/j.ajhg.2014.07.013.
37. Laššuthová, P.; Beharka, R.; Krůtová, M.; Neupauerová, J.; Seeman, P. COX6A1 Mutation Causes Axonal Hereditary Motor and Sensory Neuropathy - the Confirmation of the Primary Report. *Clin. Genet.* **2016**, *89*, 512–514, doi:10.1111/cge.12649.
38. Varon, R.; Gooding, R.; Steglich, C.; Marns, L.; Tang, H.; Angelicheva, D.; Yong, K.K.; Ambrugger, P.; Reinhold, A.; Morar, B.; et al. Partial Deficiency of the C-Terminal-Domain Phosphatase of RNA Polymerase II Is Associated with Congenital Cataracts Facial Dysmorphism Neuropathy Syndrome. *Nat. Genet.* **2003**, *35*, 185–189, doi:10.1038/ng1243.
39. Lassuthova, P.; Sišková, D.; Haberlová, J.; Sakmaryová, I.; Filouš, A.; Seeman, P. Congenital Cataract, Facial Dysmorphism and Demyelinating Neuropathy (CCFDN) in 10 Czech Gypsy Children--Frequent and Underestimated Cause of Disability among Czech Gypsies. *Orphanet J Rare Dis* **2014**, *9*, 46, doi:10.1186/1750-1172-9-46.
40. Puls, I.; Jonnakuty, C.; LaMonte, B.H.; Holzbaur, E.L.F.; Tokito, M.; Mann, E.; Floeter, M.K.; Bidus, K.; Drayna, D.; Oh, S.J.; et al. Mutant Dynactin in Motor Neuron Disease. *Nat. Genet.* **2003**, *33*, 455–456, doi:10.1038/ng1123.
41. Hwang, S.H.; Kim, E.J.; Hong, Y.B.; Joo, J.; Kim, S.M.; Nam, S.H.; Hong, H.D.; Kim, S.H.; Oh, K.; Lim, J.-G.; et al. Distal Hereditary Motor Neuropathy Type 7B with Dynactin 1 Mutation. *Mol Med Rep* **2016**, *14*, 3362–3368, doi:10.3892/mmr.2016.5664.
42. Levy, J.R.; Sumner, C.J.; Caviston, J.P.; Tokito, M.K.; Ranganathan, S.; Ligon, L.A.; Wallace, K.E.; LaMonte, B.H.; Harmison, G.G.; Puls, I.; et al. A Motor Neuron Disease-Associated Mutation in P150Glued Perturbs Dynactin Function and Induces Protein Aggregation. *J. Cell Biol.* **2006**, *172*, 733–745, doi:10.1083/jcb.200511068.
43. Braathen, G.J.; Høyer, H.; Busk, Ø.L.; Tveten, K.; Skjelbred, C.F.; Russell, M.B. Variants in the Genes DCTN2, DNAH10, LRIG3, and MYO1A Are Associated with Intermediate Charcot-Marie-Tooth Disease in a Norwegian Family. *Acta Neurol Scand* **2016**, *134*, 67–75, doi:10.1111/ane.12515.
44. Xu, W.-Y.; Gu, M.-M.; Sun, L.-H.; Guo, W.-T.; Zhu, H.-B.; Ma, J.-F.; Yuan, W.-T.; Kuang, Y.; Ji, B.-J.; Wu, X.-L.; et al. A Nonsense Mutation in DHTKD1 Causes Charcot-Marie-Tooth Disease Type 2 in a Large Chinese Pedigree. *Am. J. Hum. Genet.* **2012**, *91*, 1088–1094, doi:10.1016/j.ajhg.2012.09.018.
45. Xu, W.-Y.; Zhu, H.; Shen, Y.; Wan, Y.-H.; Tu, X.-D.; Wu, W.-T.; Tang, L.; Zhang, H.-X.; Lu, S.-Y.; Jin, X.-L.; et al. DHTKD1 Deficiency Causes Charcot-Marie-Tooth Disease in Mice. *Mol. Cell. Biol.* **2018**, *38*, doi:10.1128/MCB.00085-18.

46. Chen, C.-X.; Dong, H.-L.; Wei, Q.; Li, L.-X.; Yu, H.; Li, J.-Q.; Liu, G.-L.; Li, H.-F.; Bai, G.; Ma, H.; et al. Genetic Spectrum and Clinical Profiles in a Southeast Chinese Cohort of Charcot-Marie-Tooth Disease. *Clin Genet* **2019**, *96*, 439–448, doi:10.1111/cge.13616.
47. Hong, Y.B.; Kang, J.; Kim, J.H.; Lee, J.; Kwak, G.; Hyun, Y.S.; Nam, S.H.; Hong, H.D.; Choi, Y.-R.; Jung, S.-C.; et al. DGAT2 Mutation in a Family with Autosomal-Dominant Early-Onset Axonal Charcot-Marie-Tooth Disease. *Hum Mutat* **2016**, *37*, 473–480, doi:10.1002/humu.22959.
48. Blumen, S.C.; Astord, S.; Robin, V.; Vignaud, L.; Toumi, N.; Cieslik, A.; Achiron, A.; Carasso, R.L.; Gurevich, M.; Braverman, I.; et al. A Rare Recessive Distal Hereditary Motor Neuropathy with HSJ1 Chaperone Mutation. *Ann. Neurol.* **2012**, *71*, 509–519, doi:10.1002/ana.22684.
49. Gess, B.; Auer-Grumbach, M.; Schirmacher, A.; Strom, T.; Zitzelsberger, M.; Rudnik-Schöneborn, S.; Röhr, D.; Halfter, H.; Young, P.; Senderek, J. HSJ1-Related Hereditary Neuropathies: Novel Mutations and Extended Clinical Spectrum. *Neurology* **2014**, *83*, 1726–1732, doi:10.1212/WNL.0000000000000966.
50. Züchner, S.; Noureddine, M.; Kennerson, M.; Verhoeven, K.; Claeys, K.; De Jonghe, P.; Merory, J.; Oliveira, S.A.; Speer, M.C.; Stenger, J.E.; et al. Mutations in the Pleckstrin Homology Domain of Dynamin 2 Cause Dominant Intermediate Charcot-Marie-Tooth Disease. *Nat. Genet.* **2005**, *37*, 289–294, doi:10.1038/ng1514.
51. Fabrizi, G.M.; Ferrarini, M.; Cavallaro, T.; Cabrini, I.; Cerini, R.; Bertolasi, L.; Rizzuto, N. Two Novel Mutations in Dynamin-2 Cause Axonal Charcot-Marie-Tooth Disease. *Neurology* **2007**, *69*, 291–295, doi:10.1212/01.wnl.0000265820.51075.61.
52. Bragato, C.; Gaudenzi, G.; Blasevich, F.; Pavesi, G.; Maggi, L.; Giunta, M.; Cotelli, F.; Mora, M. Zebrafish as a Model to Investigate Dynamin 2-Related Diseases. *Sci Rep* **2016**, *6*, 20466, doi:10.1038/srep20466.
53. Klein, C.J.; Botuyan, M.-V.; Wu, Y.; Ward, C.J.; Nicholson, G.A.; Hammans, S.; Hojo, K.; Yamanishi, H.; Karpf, A.R.; Wallace, D.C.; et al. Mutations in DNMT1 Cause Hereditary Sensory Neuropathy with Dementia and Hearing Loss. *Nat. Genet.* **2011**, *43*, 595–600, doi:10.1038/ng.830.
54. Klein, C.J.; Bird, T.; Ertekin-Taner, N.; Lincoln, S.; Hjorth, R.; Wu, Y.; Kwok, J.; Mer, G.; Dyck, P.J.; Nicholson, G.A. DNMT1 Mutation Hot Spot Causes Varied Phenotypes of HSAN1 with Dementia and Hearing Loss. *Neurology* **2013**, *80*, 824–828, doi:10.1212/WNL.0b013e318284076d.
55. Brennan, K.M.; Bai, Y.; Pisciotta, C.; Wang, S.; Feely, S.M.E.; Hoegger, M.; Gutmann, L.; Moore, S.A.; Gonzalez, M.; Sherman, D.L.; et al. Absence of Dystrophin Related Protein-2 Disrupts Cajal Bands in a Patient with Charcot-Marie-Tooth Disease. *Neuromuscul Disord* **2015**, *25*, 786–793, doi:10.1016/j.nmd.2015.07.001.
56. Tahmasebi-Birgani, M.; Hajjari, M.; Golchin, N.; Shalbafan, B.; Mohammadi-Asl, J.; Sadeghian, F. Whole Exome Sequencing Revealed a Novel Dystrophin-Related Protein-2 (DRP2) Deletion in an Iranian Family with Symptoms of Polyneuropathy. *Iran J Basic Med Sci* **2019**, *22*, 576–580, doi:10.22038/ijbms.2019.30754.7414.
57. Sherman, D.L.; Brophy, P.J. A Murine Model of Charcot-Marie-Tooth Disease 4F Reveals a Role for the C-Terminus of Periaxin in the Formation and Stabilization of Cajal Bands. *Wellcome Open Res* **2018**, *3*, 20, doi:10.12688/wellcomeopenres.13673.1.
58. Edvardson, S.; Cinnamon, Y.; Jalas, C.; Shaag, A.; Maayan, C.; Axelrod, F.B.; Elpeleg, O. Hereditary Sensory Autonomic Neuropathy Caused by a Mutation in Dystonin. *Ann. Neurol.* **2012**, *71*, 569–572, doi:10.1002/ana.23524.
59. Motley, W.W.; Züchner, S.; Scherer, S.S. Isoform-Specific Loss of Dystonin Causes Hereditary Motor and Sensory Neuropathy. *Neurol Genet* **2020**, *6*, e496, doi:10.1212/NXG.0000000000000496.
60. Lynch-Godrei, A.; De Repentigny, Y.; Gagnon, S.; Trung, M.T.; Kothary, R. Dystonin-A3 Upregulation Is Responsible for Maintenance of Tubulin Acetylation in a Less Severe Dystonia Musculorum Mouse Model for Hereditary Sensory and Autonomic Neuropathy Type VI. *Hum. Mol. Genet.* **2018**, *27*, 3598–3611, doi:10.1093/hmg/ddy250.

61. Weedon, M.N.; Hastings, R.; Caswell, R.; Xie, W.; Paszkiewicz, K.; Antoniadi, T.; Williams, M.; King, C.; Greenhalgh, L.; Newbury-Ecob, R.; et al. Exome Sequencing Identifies a DYNC1H1 Mutation in a Large Pedigree with Dominant Axonal Charcot-Marie-Tooth Disease. *Am. J. Hum. Genet.* **2011**, *89*, 308–312, doi:10.1016/j.ajhg.2011.07.002.
62. Harms, M.B.; Ori-McKenney, K.M.; Scoto, M.; Tuck, E.P.; Bell, S.; Ma, D.; Masi, S.; Allred, P.; Al-Lozi, M.; Reilly, M.M.; et al. Mutations in the Tail Domain of DYNC1H1 Cause Dominant Spinal Muscular Atrophy. *Neurology* **2012**, *78*, 1714–1720, doi:10.1212/WNL.0b013e3182556c05.
63. Tsurusaki, Y.; Saitoh, S.; Tomizawa, K.; Sudo, A.; Asahina, N.; Shiraishi, H.; Ito, J.-I.; Tanaka, H.; Doi, H.; Saitsu, H.; et al. A DYNC1H1 Mutation Causes a Dominant Spinal Muscular Atrophy with Lower Extremity Predominance. *Neurogenetics* **2012**, *13*, 327–332, doi:10.1007/s10048-012-0337-6.
64. Eschbach, J.; Sinniger, J.; Bouitbir, J.; Fergani, A.; Schlagowski, A.-I.; Zoll, J.; Geny, B.; René, F.; Larmet, Y.; Marion, V.; et al. Dynein Mutations Associated with Hereditary Motor Neuropathies Impair Mitochondrial Morphology and Function with Age. *Neurobiol. Dis.* **2013**, *58*, 220–230, doi:10.1016/j.nbd.2013.05.015.
65. Warner, L.E.; Mancias, P.; Butler, I.J.; McDonald, C.M.; Keppen, L.; Koob, K.G.; Lupski, J.R. Mutations in the Early Growth Response 2 (EGR2) Gene Are Associated with Hereditary Myelinopathies. *Nat. Genet.* **1998**, *18*, 382–384, doi:10.1038/ng0498-382.
66. Timmerman, V.; De Jonghe, P.; Ceuterick, C.; De Vriendt, E.; Löfgren, A.; Nelis, E.; Warner, L.E.; Lupski, J.R.; Martin, J.J.; Van Broeckhoven, C. Novel Missense Mutation in the Early Growth Response 2 Gene Associated with Dejerine-Sottas Syndrome Phenotype. *Neurology* **1999**, *52*, 1827–1832, doi:10.1212/wnl.52.9.1827.
67. Topilko, P.; Schneider-Maunoury, S.; Levi, G.; Baron-Van Evercooren, A.; Chennoufi, A.B.; Seitanidou, T.; Babinet, C.; Charnay, P. Krox-20 Controls Myelination in the Peripheral Nervous System. *Nature* **1994**, *371*, 796–799, doi:10.1038/371796a0.
68. Slaugenhaupt, S.A.; Blumenfeld, A.; Gill, S.P.; Leyne, M.; Mull, J.; Cuajungco, M.P.; Liebert, C.B.; Chadwick, B.; Idelson, M.; Reznik, L.; et al. Tissue-Specific Expression of a Splicing Mutation in the IKBKAP Gene Causes Familial Dysautonomia. *Am. J. Hum. Genet.* **2001**, *68*, 598–605, doi:10.1086/318810.
69. Leyne, M.; Mull, J.; Gill, S.P.; Cuajungco, M.P.; Oddoux, C.; Blumenfeld, A.; Maayan, C.; Gusella, J.F.; Axelrod, F.B.; Slaugenhaupt, S.A. Identification of the First Non-Jewish Mutation in Familial Dysautonomia. *Am. J. Med. Genet. A* **2003**, *118A*, 305–308, doi:10.1002/ajmg.a.20052.
70. Hims, M.M.; Shetty, R.S.; Pickel, J.; Mull, J.; Leyne, M.; Liu, L.; Gusella, J.F.; Slaugenhaupt, S.A. A Humanized IKBKAP Transgenic Mouse Models a Tissue-Specific Human Splicing Defect. *Genomics* **2007**, *90*, 389–396, doi:10.1016/j.ygeno.2007.05.012.
71. Auer-Grumbach, M.; Weger, M.; Fink-Puches, R.; Papić, L.; Fröhlich, E.; Auer-Grumbach, P.; El Shabrawi-Caelen, L.; Schabhüttl, M.; Windpassinger, C.; Senderek, J.; et al. Fibulin-5 Mutations Link Inherited Neuropathies, Age-Related Macular Degeneration and Hyperelastic Skin. *Brain* **2011**, *134*, 1839–1852, doi:10.1093/brain/awr076.
72. Šafka Brozková, D.; Laššuthová, P.; Neupauerová, J.; Krůtová, M.; Haberlová, J.; Stejskal, D.; Seeman, P. Czech Family Confirms the Link between FBLN5 and Charcot-Marie-Tooth Type 1 Neuropathy. *Brain* **2013**, *136*, e232, doi:10.1093/brain/aws333.
73. Cheng, S.; Lv, H.; Zhang, W.; Wang, Z.; Shi, X.; Liang, W.; Yuan, Y. Adult-Onset Demyelinating Neuropathy Associated with FBLN5 Gene Mutation. *Clin. Neuropathol.* **2017**, *36*, 171–177, doi:10.5414/NP301011.
74. Won, S.Y.; Kwon, S.; Jeong, H.S.; Chung, K.W.; Choi, B.-O.; Chang, J.W.; Lee, J.E. Fibulin 5, a Human Wharton's Jelly-Derived Mesenchymal Stem Cells-Secreted Paracrine Factor, Attenuates Peripheral Nervous System Myelination Defects through the Integrin-RAC1 Signaling Axis. *Stem Cells* **2020**, doi:10.1002/stem.3287.
75. Sumner, C.J.; d'Ydewalle, C.; Wooley, J.; Fawcett, K.A.; Hernandez, D.; Gardiner, A.R.; Kalmar, B.; Baloh, R.H.; Gonzalez, M.; Züchner, S.; et al. A Dominant Mutation in FBXO38 Causes Distal Spinal Muscular Atrophy with Calf Predominance. *Am. J. Hum. Genet.* **2013**, *93*, 976–983, doi:10.1016/j.ajhg.2013.10.006.

76. Akçimen, F.; Vural, A.; Durmuş, H.; Çakar, A.; Houlden, H.; Parman, Y.G.; Nazlı Başak, A. A Novel Homozygous FBXO38 Variant Causes an Early-Onset Distal Hereditary Motor Neuronopathy Type IID. *J. Hum. Genet.* **2019**, *64*, 1141–1144, doi:10.1038/s10038-019-0652-y.
77. De Sandre-Giovannoli, A.; Delague, V.; Hamadouche, T.; Chaouch, M.; Krahn, M.; Boccaccio, I.; Maisonobe, T.; Chouery, E.; Jabbour, R.; Atweh, S.; et al. Homozygosity Mapping of Autosomal Recessive Demyelinating Charcot-Marie-Tooth Neuropathy (CMT4H) to a Novel Locus on Chromosome 12p11.21-Q13.11. *J. Med. Genet.* **2005**, *42*, 260–265, doi:10.1136/jmg.2004.024364.
78. Stendel, C.; Roos, A.; Deconinck, T.; Pereira, J.; Castagner, F.; Niemann, A.; Kirschner, J.; Korinthenberg, R.; Ketelsen, U.-P.; Battaloglu, E.; et al. Peripheral Nerve Demyelination Caused by a Mutant Rho GTPase Guanine Nucleotide Exchange Factor, Frabin/FGD4. *Am. J. Hum. Genet.* **2007**, *81*, 158–164, doi:10.1086/518770.
79. Delague, V.; Jacquier, A.; Hamadouche, T.; Poitelon, Y.; Baudot, C.; Boccaccio, I.; Chouery, E.; Chaouch, M.; Kassouri, N.; Jabbour, R.; et al. Mutations in FGD4 Encoding the Rho GDP/GTP Exchange Factor FRABIN Cause Autosomal Recessive Charcot-Marie-Tooth Type 4H. *Am. J. Hum. Genet.* **2007**, *81*, 1–16, doi:10.1086/518428.
80. Chow, C.Y.; Zhang, Y.; Dowling, J.J.; Jin, N.; Adamska, M.; Shiga, K.; Szigeti, K.; Shy, M.E.; Li, J.; Zhang, X.; et al. Mutation of FIG4 Causes Neurodegeneration in the Pale Tremor Mouse and Patients with CMT4J. *Nature* **2007**, *448*, 68–72, doi:10.1038/nature05876.
81. Nicholson, G.; Lenk, G.M.; Reddel, S.W.; Grant, A.E.; Towne, C.F.; Ferguson, C.J.; Simpson, E.; Scheuerle, A.; Yasick, M.; Hoffman, S.; et al. Distinctive Genetic and Clinical Features of CMT4J: A Severe Neuropathy Caused by Mutations in the PI(3,5)P_2 Phosphatase FIG4. *Brain* **2011**, *134*, 1959–1971, doi:10.1093/brain/awr148.
82. Zhang, X.; Chow, C.Y.; Sahenk, Z.; Shy, M.E.; Meisler, M.H.; Li, J. Mutation of FIG4 Causes a Rapidly Progressive, Asymmetric Neuronal Degeneration. *Brain* **2008**, *131*, 1990–2001, doi:10.1093/brain/awn114.
83. Ionasescu, V.; Searby, C.; Sheffield, V.C.; Roklina, T.; Nishimura, D.; Ionasescu, R. Autosomal Dominant Charcot-Marie-Tooth Axonal Neuropathy Mapped on Chromosome 7p (CMT2D). *Hum. Mol. Genet.* **1996**, *5*, 1373–1375, doi:10.1093/hmg/5.9.1373.
84. Antonellis, A.; Ellsworth, R.E.; Sambuughin, N.; Puls, I.; Abel, A.; Lee-Lin, S.-Q.; Jordanova, A.; Kremensky, I.; Christodoulou, K.; Middleton, L.T.; et al. Glycyl TRNA Synthetase Mutations in Charcot-Marie-Tooth Disease Type 2D and Distal Spinal Muscular Atrophy Type V. *Am. J. Hum. Genet.* **2003**, *72*, 1293–1299, doi:10.1086/375039.
85. Griffin, L.B.; Sakaguchi, R.; McGuigan, D.; Gonzalez, M.A.; Searby, C.; Züchner, S.; Hou, Y.-M.; Antonellis, A. Impaired Function Is a Common Feature of Neuropathy-Associated Glycyl-TRNA Synthetase Mutations. *Hum. Mutat.* **2014**, *35*, 1363–1371, doi:10.1002/humu.22681.
86. He, W.; Bai, G.; Zhou, H.; Wei, N.; White, N.M.; Lauer, J.; Liu, H.; Shi, Y.; Dumitru, C.D.; Lettieri, K.; et al. CMT2D Neuropathy Is Linked to the Neomorphic Binding Activity of Glycyl-TRNA Synthetase. *Nature* **2015**, *526*, 710–714, doi:10.1038/nature15510.
87. Mendoza-Ferreira, N.; Karakaya, M.; Cengiz, N.; Beijer, D.; Brigatti, K.W.; Gonzaga-Jauregui, C.; Fuhrmann, N.; Hölker, I.; Thelen, M.P.; Zetzsche, S.; et al. De Novo and Inherited Variants in GBF1 Are Associated with Axonal Neuropathy Caused by Golgi Fragmentation. *Am J Hum Genet* **2020**, *107*, 763–777, doi:10.1016/j.ajhg.2020.08.018.
88. Baxter, R.V.; Ben Othmane, K.; Rochelle, J.M.; Stajich, J.E.; Hulette, C.; Dew-Knight, S.; Hentati, F.; Ben Hamida, M.; Bel, S.; Stenger, J.E.; et al. Ganglioside-Induced Differentiation-Associated Protein-1 Is Mutant in Charcot-Marie-Tooth Disease Type 4A/8q21. *Nat. Genet.* **2002**, *30*, 21–22, doi:10.1038/ng796.
89. Claramunt, R.; Pedrola, L.; Sevilla, T.; López de Munain, A.; Berciano, J.; Cuesta, A.; Sánchez-Navarro, B.; Millán, J.M.; Saifi, G.M.; Lupski, J.R.; et al. Genetics of Charcot-Marie-Tooth Disease Type 4A: Mutations, Inheritance, Phenotypic Variability, and Founder Effect. *J. Med. Genet.* **2005**, *42*, 358–365, doi:10.1136/jmg.2004.022178.

90. Zimoń, M.; Baets, J.; Fabrizi, G.M.; Jaakkola, E.; Kabzińska, D.; Pilch, J.; Schindler, A.B.; Cornblath, D.R.; Fischbeck, K.H.; Auer-Grumbach, M.; et al. Dominant GDAP1 Mutations Cause Predominantly Mild CMT Phenotypes. *Neurology* **2011**, *77*, 540–548, doi:10.1212/WNL.0b013e318228fc70.
91. Bergoffen, J.; Scherer, S.S.; Wang, S.; Scott, M.O.; Bone, L.J.; Paul, D.L.; Chen, K.; Lensch, M.W.; Chance, P.F.; Fischbeck, K.H. Connexin Mutations in X-Linked Charcot-Marie-Tooth Disease. *Science* **1993**, *262*, 2039–2042, doi:10.1126/science.8266101.
92. Latour, P.; Lévy, N.; Paret, M.; Chapon, F.; Chazot, G.; Clavelou, P.; Couratier, P.; Dumas, R.; Ollagnon, E.; Pouget, J.; et al. Mutations in the X-Linked Form of Charcot-Marie-Tooth Disease in the French Population. *Neurogenetics* **1997**, *1*, 117–123, doi:10.1007/s100480050017.
93. Liang, G.S.L.; de Miguel, M.; Gómez-Hernández, J.M.; Glass, J.D.; Scherer, S.S.; Mintz, M.; Barrio, L.C.; Fischbeck, K.H. Severe Neuropathy with Leaky Connexin32 Hemichannels. *Ann. Neurol.* **2005**, *57*, 749–754, doi:10.1002/ana.20459.
94. Wang, W.; Wang, C.; Dawson, D.B.; Thorland, E.C.; Lundquist, P.A.; Eckloff, B.W.; Wu, Y.; Baheti, S.; Evans, J.M.; Scherer, S.S.; et al. Target-Enrichment Sequencing and Copy Number Evaluation in Inherited Polyneuropathy. *Neurology* **2016**, *86*, 1762–1771, doi:10.1212/WNL.0000000000002659.
95. Garman, S.C.; Garboczi, D.N. Structural Basis of Fabry Disease. *Mol Genet Metab* **2002**, *77*, 3–11, doi:10.1016/s1096-7192(02)00151-8.
96. Soong, B.-W.; Huang, Y.-H.; Tsai, P.-C.; Huang, C.-C.; Pan, H.-C.; Lu, Y.-C.; Chien, H.-J.; Liu, T.-T.; Chang, M.-H.; Lin, K.-P.; et al. Exome Sequencing Identifies GNB4 Mutations as a Cause of Dominant Intermediate Charcot-Marie-Tooth Disease. *Am. J. Hum. Genet.* **2013**, *92*, 422–430, doi:10.1016/j.ajhg.2013.01.014.
97. Laššuthová, P.; Šafka Brožková, D.; Neupauerová, J.; Krůtová, M.; Mazanec, R.; Seeman, P. Confirmation of the GNB4 Gene as Causal for Charcot-Marie-Tooth Disease by a Novel de Novo Mutation in a Czech Patient. *Neuromuscul. Disord.* **2017**, *27*, 57–60, doi:10.1016/j.nmd.2016.09.010.
98. Vester, A.; Velez-Ruiz, G.; McLaughlin, H.M.; NISC Comparative Sequencing Program; Lupski, J.R.; Talbot, K.; Vance, J.M.; Züchner, S.; Roda, R.H.; Fischbeck, K.H.; et al. A Loss-of-Function Variant in the Human Histidyl-TRNA Synthetase (HARS) Gene Is Neurotoxic in Vivo. *Hum. Mutat.* **2013**, *34*, 191–199, doi:10.1002/humu.22210.
99. Safka Brozkova, D.; Deconinck, T.; Griffin, L.B.; Ferbert, A.; Haberlova, J.; Mazanec, R.; Lassuthova, P.; Roth, C.; Pilunthanakul, T.; Rautenstrauss, B.; et al. Loss of Function Mutations in HARS Cause a Spectrum of Inherited Peripheral Neuropathies. *Brain* **2015**, *138*, 2161–2172, doi:10.1093/brain/awv158.
100. Zimoń, M.; Baets, J.; Almeida-Souza, L.; De Vriendt, E.; Nikodinovic, J.; Parman, Y.; Battaloğlu, E.; Matur, Z.; Guergueltcheva, V.; Tournev, I.; et al. Loss-of-Function Mutations in HINT1 Cause Axonal Neuropathy with Neuromyotonia. *Nat. Genet.* **2012**, *44*, 1080–1083, doi:10.1038/ng.2406.
101. Laššuthová, P.; Brožková, D.Š.; Krůtová, M.; Neupauerová, J.; Haberlová, J.; Mazanec, R.; Dvořáčková, N.; Goldenberg, Z.; Seeman, P. Mutations in HINT1 Are One of the Most Frequent Causes of Hereditary Neuropathy among Czech Patients and Neuromyotonia Is Rather an Underdiagnosed Symptom. *Neurogenetics* **2015**, *16*, 43–54, doi:10.1007/s10048-014-0427-8.
102. Hantke, J.; Chandler, D.; King, R.; Wanders, R.J.A.; Angelicheva, D.; Tournev, I.; McNamara, E.; Kwa, M.; Guergueltcheva, V.; Kaneva, R.; et al. A Mutation in an Alternative Untranslated Exon of Hexokinase 1 Associated with Hereditary Motor and Sensory Neuropathy -- Russe (HMSNR). *Eur. J. Hum. Genet.* **2009**, *17*, 1606–1614, doi:10.1038/ejhg.2009.99.
103. Sevilla, T.; Martínez-Rubio, D.; Márquez, C.; Paradas, C.; Colomer, J.; Jaijo, T.; Millán, J.M.; Palau, F.; Espinós, C. Genetics of the Charcot-Marie-Tooth Disease in the Spanish Gypsy Population: The Hereditary Motor and Sensory Neuropathy-Russe in Depth. *Clin. Genet.* **2013**, *83*, 565–570, doi:10.1111/cge.12015.
104. Evgrafov, O.V.; Mersiyanova, I.; Irobi, J.; Van Den Bosch, L.; Dierick, I.; Leung, C.L.; Schagina, O.; Verpoorten, N.; Van Impe, K.; Fedotov, V.; et al. Mutant Small Heat-Shock Protein 27 Causes Axonal Charcot-Marie-Tooth Disease and Distal Hereditary Motor Neuropathy. *Nat. Genet.* **2004**, *36*, 602–606, doi:10.1038/ng1354.
105. Houlden, H.; Laura, M.; Wavrant-De Vrièze, F.; Blake, J.; Wood, N.; Reilly, M.M. Mutations in the HSP27 (HSPB1) Gene Cause Dominant, Recessive, and Sporadic Distal HMN/CMT Type 2. *Neurology* **2008**, *71*, 1660–1668, doi:10.1212/01.wnl.0000319696.14225.67.

106. d'Ydewalle, C.; Krishnan, J.; Chiheb, D.M.; Van Damme, P.; Irobi, J.; Kozikowski, A.P.; Vanden Berghe, P.; Timmerman, V.; Robberecht, W.; Van Den Bosch, L. HDAC6 Inhibitors Reverse Axonal Loss in a Mouse Model of Mutant HSPB1-Induced Charcot-Marie-Tooth Disease. *Nat. Med.* **2011**, *17*, 968–974, doi:10.1038/nm.2396.
107. Kolb, S.J.; Snyder, P.J.; Poi, E.J.; Renard, E.A.; Bartlett, A.; Gu, S.; Sutton, S.; Arnold, W.D.; Freimer, M.L.; Lawson, V.H.; et al. Mutant Small Heat Shock Protein B3 Causes Motor Neuropathy: Utility of a Candidate Gene Approach. *Neurology* **2010**, *74*, 502–506, doi:10.1212/WNL.0b013e3181cef84a.
108. Nam, D.E.; Nam, S.H.; Lee, A.J.; Hong, Y.B.; Choi, B.-O.; Chung, K.W. Small Heat Shock Protein B3 (HSPB3) Mutation in an Axonal Charcot-Marie-Tooth Disease Family. *J. Peripher. Nerv. Syst.* **2018**, *23*, 60–66, doi:10.1111/jns.12249.
109. La Padula, V.; Staszewski, O.; Nestel, S.; Busch, H.; Boerries, M.; Roussa, E.; Prinz, M.; Krieglstein, K. HSPB3 Protein Is Expressed in Motoneurons and Induces Their Survival after Lesion-Induced Degeneration. *Exp. Neurol.* **2016**, *286*, 40–49, doi:10.1016/j.expneurol.2016.08.014.
110. Irobi, J.; Van Impe, K.; Seeman, P.; Jordanova, A.; Dierick, I.; Verpoorten, N.; Michalik, A.; De Vriendt, E.; Jacobs, A.; Van Gerwen, V.; et al. Hot-Spot Residue in Small Heat-Shock Protein 22 Causes Distal Motor Neuropathy. *Nat. Genet.* **2004**, *36*, 597–601, doi:10.1038/ng1328.
111. Tang, B.; Zhao, G.; Luo, W.; Xia, K.; Cai, F.; Pan, Q.; Zhang, R.; Zhang, F.; Liu, X.; Chen, B.; et al. Small Heat-Shock Protein 22 Mutated in Autosomal Dominant Charcot-Marie-Tooth Disease Type 2L. *Hum. Genet.* **2005**, *116*, 222–224, doi:10.1007/s00439-004-1218-3.
112. Irobi, J.; Almeida-Souza, L.; Asselbergh, B.; De Winter, V.; Goethals, S.; Dierick, I.; Krishnan, J.; Timmermans, J.-P.; Robberecht, W.; De Jonghe, P.; et al. Mutant HSPB8 Causes Motor Neuron-Specific Neurite Degeneration. *Hum. Mol. Genet.* **2010**, *19*, 3254–3265, doi:10.1093/hmg/ddq234.
113. Cottenie, E.; Kochanski, A.; Jordanova, A.; Bansagi, B.; Zimon, M.; Horga, A.; Jaunmuktane, Z.; Saveri, P.; Rasic, V.M.; Baets, J.; et al. Truncating and Missense Mutations in IGHMBP2 Cause Charcot-Marie Tooth Disease Type 2. *Am. J. Hum. Genet.* **2014**, *95*, 590–601, doi:10.1016/j.ajhg.2014.10.002.
114. Schottmann, G.; Jungbluth, H.; Schara, U.; Knierim, E.; Morales Gonzalez, S.; Gill, E.; Seifert, F.; Norwood, F.; Deshpande, C.; von Au, K.; et al. Recessive Truncating IGHMBP2 Mutations Presenting as Axonal Sensorimotor Neuropathy. *Neurology* **2015**, *84*, 523–531, doi:10.1212/WNL.0000000000001220.
115. Boyer, O.; Nevo, F.; Plaisier, E.; Funalot, B.; Gribouval, O.; Benoit, G.; Huynh Cong, E.; Arrondel, C.; Tête, M.-J.; Montjean, R.; et al. INF2 Mutations in Charcot-Marie-Tooth Disease with Glomerulopathy. *N. Engl. J. Med.* **2011**, *365*, 2377–2388, doi:10.1056/NEJMoa1109122.
116. Echaniz-Laguna, A.; Latour, P. A Cryptic Splicing Mutation in the INF2 Gene Causing Charcot-Marie-Tooth Disease with Minimal Glomerular Dysfunction. *J. Peripher. Nerv. Syst.* **2019**, *24*, 120–124, doi:10.1111/jns.12308.
117. Rönkkö, J.; Molchanova, S.; Revah-Politi, A.; Pereira, E.M.; Auranen, M.; Toppila, J.; Kvist, J.; Ludwig, A.; Neumann, J.; Bultynck, G.; et al. Dominant Mutations in ITPR3 Cause Charcot-Marie-Tooth Disease. *Ann Clin Transl Neurol* **2020**, *7*, 1962–1972, doi:10.1002/acn3.51190.
118. Sullivan, J.M.; Motley, W.W.; Johnson, J.O.; Aisenberg, W.H.; Marshall, K.L.; Barwick, K.E.; Kong, L.; Huh, J.S.; Saavedra-Rivera, P.C.; McEntagart, M.M.; et al. Dominant Mutations of the Notch Ligand Jagged1 Cause Peripheral Neuropathy. *J Clin Invest* **2020**, *130*, 1506–1512, doi:10.1172/JCI128152.
119. McLaughlin, H.M.; Sakaguchi, R.; Liu, C.; Igarashi, T.; Pehlivan, D.; Chu, K.; Iyer, R.; Cruz, P.; Cherukuri, P.F.; Hansen, N.F.; et al. Compound Heterozygosity for Loss-of-Function Lysyl-TRNA Synthetase Mutations in a Patient with Peripheral Neuropathy. *Am. J. Hum. Genet.* **2010**, *87*, 560–566, doi:10.1016/j.ajhg.2010.09.008.
120. Rivière, J.-B.; Ramalingam, S.; Lavastre, V.; Shekarabi, M.; Holbert, S.; Lafontaine, J.; Srour, M.; Merner, N.; Rochefort, D.; Hince, P.; et al. KIF1A, an Axonal Transporter of Synaptic Vesicles, Is Mutated in Hereditary Sensory and Autonomic Neuropathy Type 2. *Am. J. Hum. Genet.* **2011**, *89*, 219–230, doi:10.1016/j.ajhg.2011.06.013.
121. Klebe, S.; Lossos, A.; Azzedine, H.; Mundwiller, E.; Sheffer, R.; Gaussen, M.; Marelli, C.; Nawara, M.; Carpentier, W.; Meyer, V.; et al. KIF1A Missense Mutations in SPG30, an Autosomal Recessive Spastic Paraplegia: Distinct Phenotypes According to the Nature of the Mutations. *Eur. J. Hum. Genet.* **2012**, *20*, 645–649, doi:10.1038/ejhg.2011.261.

122. Tanaka, Y.; Niwa, S.; Dong, M.; Farkhondeh, A.; Wang, L.; Zhou, R.; Hirokawa, N. The Molecular Motor KIF1A Transports the TrkA Neurotrophin Receptor and Is Essential for Sensory Neuron Survival and Function. *Neuron* **2016**, *90*, 1215–1229, doi:10.1016/j.neuron.2016.05.002.
123. Zhao, C.; Takita, J.; Tanaka, Y.; Setou, M.; Nakagawa, T.; Takeda, S.; Yang, H.W.; Terada, S.; Nakata, T.; Takei, Y.; et al. Charcot-Marie-Tooth Disease Type 2A Caused by Mutation in a Microtubule Motor KIF1Bbeta. *Cell* **2001**, *105*, 587–597, doi:10.1016/s0092-8674(01)00363-4.
124. Drew, A.P.; Zhu, D.; Kidambi, A.; Ly, C.; Tey, S.; Brewer, M.H.; Ahmad-Annuar, A.; Nicholson, G.A.; Kennerson, M.L. Improved Inherited Peripheral Neuropathy Genetic Diagnosis by Whole-Exome Sequencing. *Mol Genet Genomic Med* **2015**, *3*, 143–154, doi:10.1002/mgg3.126.
125. Lyons, D.A.; Naylor, S.G.; Scholze, A.; Talbot, W.S. Kif1b Is Essential for MRNA Localization in Oligodendrocytes and Development of Myelinated Axons. *Nat. Genet.* **2009**, *41*, 854–858, doi:10.1038/ng.376.
126. Xu, F.; Takahashi, H.; Tanaka, Y.; Ichinose, S.; Niwa, S.; Wicklund, M.P.; Hirokawa, N. KIF1Bβ Mutations Detected in Hereditary Neuropathy Impair IGF1R Transport and Axon Growth. *J. Cell Biol.* **2018**, *217*, 3480–3496, doi:10.1083/jcb.201801085.
127. Liu, Y.-T.; Laurá, M.; Hersheson, J.; Horga, A.; Jaunmuktane, Z.; Brandner, S.; Pittman, A.; Hughes, D.; Polke, J.M.; Sweeney, M.G.; et al. Extended Phenotypic Spectrum of KIF5A Mutations: From Spastic Paraplegia to Axonal Neuropathy. *Neurology* **2014**, *83*, 612–619, doi:10.1212/WNL.0000000000000691.
128. Nam, D.E.; Yoo, D.H.; Choi, S.S.; Choi, B.-O.; Chung, K.W. Wide Phenotypic Spectrum in Axonal Charcot-Marie-Tooth Neuropathy Type 2 Patients with KIF5A Mutations. *Genes Genomics* **2018**, *40*, 77–84, doi:10.1007/s13258-017-0612-x.
129. Street, V.A.; Bennett, C.L.; Goldy, J.D.; Shirk, A.J.; Kleopa, K.A.; Tempel, B.L.; Lipe, H.P.; Scherer, S.S.; Bird, T.D.; Chance, P.F. Mutation of a Putative Protein Degradation Gene LITAF/SIMPLE in Charcot-Marie-Tooth Disease 1C. *Neurology* **2003**, *60*, 22–26, doi:10.1212/wnl.60.1.22.
130. Saifi, G.M.; Szigeti, K.; Wiszniewski, W.; Shy, M.E.; Krajewski, K.; Hausmanowa-Petrusewicz, I.; Kochanski, A.; Reeser, S.; Mancias, P.; Butler, I.; et al. SIMPLE Mutations in Charcot-Marie-Tooth Disease and the Potential Role of Its Protein Product in Protein Degradation. *Hum. Mutat.* **2005**, *25*, 372–383, doi:10.1002/humu.20153.
131. Lee, S.M.; Sha, D.; Mohammed, A.A.; Asress, S.; Glass, J.D.; Chin, L.-S.; Li, L. Motor and Sensory Neuropathy Due to Myelin Infolding and Paranodal Damage in a Transgenic Mouse Model of Charcot-Marie-Tooth Disease Type 1C. *Hum. Mol. Genet.* **2013**, *22*, 1755–1770, doi:10.1093/hmg/ddt022.
132. De Sandre-Giovannoli, A.; Chaouch, M.; Kozlov, S.; Vallat, J.-M.; Tazir, M.; Kassouri, N.; Szepetowski, P.; Hammadouche, T.; Vandenberghe, A.; Stewart, C.L.; et al. Homozygous Defects in LMNA, Encoding Lamin A/C Nuclear-Envelope Proteins, Cause Autosomal Recessive Axonal Neuropathy in Human (Charcot-Marie-Tooth Disorder Type 2) and Mouse. *Am. J. Hum. Genet.* **2002**, *70*, 726–736, doi:10.1086/339274.
133. Ben Yaou, R.; Toutain, A.; Arimura, T.; Demay, L.; Massart, C.; Peccate, C.; Muchir, A.; Llense, S.; Deburgrave, N.; Leturcq, F.; et al. Multitissular Involvement in a Family with LMNA and EMD Mutations: Role of Digenic Mechanism? *Neurology* **2007**, *68*, 1883–1894, doi:10.1212/01.wnl.0000263138.57257.6a.
134. Guernsey, D.L.; Jiang, H.; Bedard, K.; Evans, S.C.; Ferguson, M.; Matsuoka, M.; Macgillivray, C.; Nightingale, M.; Perry, S.; Rideout, A.L.; et al. Mutation in the Gene Encoding Ubiquitin Ligase LRSAM1 in Patients with Charcot-Marie-Tooth Disease. *PLoS Genet.* **2010**, *6*, doi:10.1371/journal.pgen.1001081.
135. Peeters, K.; Palaima, P.; Pelayo-Negro, A.L.; García, A.; Gallardo, E.; García-Barredo, R.; Mateiu, L.; Baets, J.; Menten, B.; De Vriendt, E.; et al. Charcot-Marie-Tooth Disease Type 2G Redefined by a Novel Mutation in LRSAM1. *Ann. Neurol.* **2016**, *80*, 823–833, doi:10.1002/ana.24775.
136. Weterman, M.A.J.; Sorrentino, V.; Kasher, P.R.; Jakobs, M.E.; van Engelen, B.G.M.; Fluiter, K.; de Wissel, M.B.; Sizarov, A.; Nürnberg, G.; Nürnberg, P.; et al. A Frameshift Mutation in LRSAM1 Is Responsible for a Dominant Hereditary Polyneuropathy. *Hum. Mol. Genet.* **2012**, *21*, 358–370, doi:10.1093/hmg/ddr471.
137. Gonzalez, M.; McLaughlin, H.; Houlden, H.; Guo, M.; Yo-Tsen, L.; Hadjivassilious, M.; Speziani, F.; Yang, X.-L.; Antonellis, A.; Reilly, M.M.; et al. Exome Sequencing Identifies a Significant Variant in Methionyl-TRNA Synthetase (MARS) in a Family with Late-Onset CMT2. *J. Neurol. Neurosurg. Psychiatry* **2013**, *84*, 1247–1249, doi:10.1136/jnnp-2013-305049.

138. Hyun, Y.S.; Park, H.J.; Heo, S.-H.; Yoon, B.R.; Nam, S.H.; Kim, S.-B.; Park, C.I.; Choi, B.-O.; Chung, K.W. Rare Variants in Methionyl- and Tyrosyl-TRNA Synthetase Genes in Late-Onset Autosomal Dominant Charcot-Marie-Tooth Neuropathy. *Clin. Genet.* **2014**, *86*, 592–594, doi:10.1111/cge.12327.
139. Gillespie, M.K.; McMillan, H.J.; Kernohan, K.D.; Pena, I.A.; Meyer-Schuman, R.; Care4Rare Canada Consortium; Antonellis, A.; Boycott, K.M. A Novel Mutation in MARS in a Patient with Charcot-Marie-Tooth Disease, Axonal, Type 2U with Congenital Onset. *J Neuromuscul Dis* **2019**, *6*, 333–339, doi:10.3233/JND-190404.
140. Leal, A.; Huehne, K.; Bauer, F.; Sticht, H.; Berger, P.; Suter, U.; Morera, B.; Del Valle, G.; Lupski, J.R.; Ekici, A.; et al. Identification of the Variant Ala335Val of MED25 as Responsible for CMT2B2: Molecular Data, Functional Studies of the SH3 Recognition Motif and Correlation between Wild-Type MED25 and PMP22 RNA Levels in CMT1A Animal Models. *Neurogenetics* **2009**, *10*, 275–287, doi:10.1007/s10048-009-0183-3.
141. Leal, A.; Bogantes-Ledezma, S.; Ekici, A.B.; Uebe, S.; Thiel, C.T.; Sticht, H.; Berghoff, M.; Berghoff, C.; Morera, B.; Meisterernst, M.; et al. The Polynucleotide Kinase 3'-Phosphatase Gene (PNKP) Is Involved in Charcot-Marie-Tooth Disease (CMT2B2) Previously Related to MED25. *Neurogenetics* **2018**, *19*, 215–225, doi:10.1007/s10048-018-0555-7.
142. Züchner, S.; Mersiyanova, I.V.; Muglia, M.; Bissar-Tadmouri, N.; Rochelle, J.; Dadali, E.L.; Zappia, M.; Nelis, E.; Patitucci, A.; Senderek, J.; et al. Mutations in the Mitochondrial GTPase Mitofusin 2 Cause Charcot-Marie-Tooth Neuropathy Type 2A. *Nat. Genet.* **2004**, *36*, 449–451, doi:10.1038/ng1341.
143. Chung, K.W.; Kim, S.B.; Park, K.D.; Choi, K.G.; Lee, J.H.; Eun, H.W.; Suh, J.S.; Hwang, J.H.; Kim, W.K.; Seo, B.C.; et al. Early Onset Severe and Late-Onset Mild Charcot-Marie-Tooth Disease with Mitofusin 2 (MFN2) Mutations. *Brain* **2006**, *129*, 2103–2118, doi:10.1093/brain/awl174.
144. Detmer, S.A.; Vande Velde, C.; Cleveland, D.W.; Chan, D.C. Hindlimb Gait Defects Due to Motor Axon Loss and Reduced Distal Muscles in a Transgenic Mouse Model of Charcot-Marie-Tooth Type 2A. *Hum. Mol. Genet.* **2008**, *17*, 367–375, doi:10.1093/hmg/ddm314.
145. Higuchi, Y.; Hashiguchi, A.; Yuan, J.; Yoshimura, A.; Mitsui, J.; Ishiura, H.; Tanaka, M.; Ishihara, S.; Tanabe, H.; Nozuma, S.; et al. Mutations in MME Cause an Autosomal-Recessive Charcot-Marie-Tooth Disease Type 2. *Ann. Neurol.* **2016**, *79*, 659–672, doi:10.1002/ana.24612.
146. Auer-Grumbach, M.; Toegel, S.; Schabhüttl, M.; Weinmann, D.; Chiari, C.; Bennett, D.L.H.; Beetz, C.; Klein, D.; Andersen, P.M.; Böhme, I.; et al. Rare Variants in MME, Encoding Metalloprotease Neprilysin, Are Linked to Late-Onset Autosomal-Dominant Axonal Polyneuropathies. *Am. J. Hum. Genet.* **2016**, *99*, 607–623, doi:10.1016/j.ajhg.2016.07.008.
147. Sevilla, T.; Lupo, V.; Martínez-Rubio, D.; Sancho, P.; Sivera, R.; Chumillas, M.J.; García-Romero, M.; Pascual-Pascual, S.I.; Muelas, N.; Dopazo, J.; et al. Mutations in the MORC2 Gene Cause Axonal Charcot-Marie-Tooth Disease. *Brain* **2016**, *139*, 62–72, doi:10.1093/brain/awv311.
148. Albulym, O.M.; Kennerson, M.L.; Harms, M.B.; Drew, A.P.; Siddell, A.H.; Auer-Grumbach, M.; Pestronk, A.; Connolly, A.; Baloh, R.H.; Zuchner, S.; et al. MORC2 Mutations Cause Axonal Charcot-Marie-Tooth Disease with Pyramidal Signs. *Ann. Neurol.* **2016**, *79*, 419–427, doi:10.1002/ana.24575.
149. Sancho, P.; Bartesaghi, L.; Miossec, O.; García-García, F.; Ramírez-Jiménez, L.; Siddell, A.; Åkesson, E.; Hedlund, E.; Laššuthová, P.; Pascual-Pascual, S.I.; et al. Characterization of Molecular Mechanisms Underlying the Axonal Charcot-Marie-Tooth Neuropathy Caused by MORC2 Mutations. *Hum. Mol. Genet.* **2019**, *28*, 1629–1644, doi:10.1093/hmg/ddz006.
150. Choi, Y.-R.; Hong, Y.B.; Jung, S.-C.; Lee, J.H.; Kim, Y.J.; Park, H.J.; Lee, J.; Koo, H.; Lee, J.-S.; Jwa, D.H.; et al. A Novel Homozygous MPV17 Mutation in Two Families with Axonal Sensorimotor Polyneuropathy. *BMC Neurol* **2015**, *15*, 179, doi:10.1186/s12883-015-0430-1.
151. Baumann, M.; Schreiber, H.; Schlotter-Weigel, B.; Löscher, W.N.; Stucka, R.; Karall, D.; Strom, T.M.; Bauer, P.; Krabichler, B.; Fauth, C.; et al. MPV17 Mutations in Juvenile- and Adult-Onset Axonal Sensorimotor Polyneuropathy. *Clin Genet* **2019**, *95*, 182–186, doi:10.1111/cge.13462.
152. Hayasaka, K.; Himoro, M.; Sato, W.; Takada, G.; Uyemura, K.; Shimizu, N.; Bird, T.D.; Conneally, P.M.; Chance, P.F. Charcot-Marie-Tooth Neuropathy Type 1B Is Associated with Mutations of the Myelin P0 Gene. *Nat. Genet.* **1993**, *5*, 31–34, doi:10.1038/ng0993-31.

153. Mandich, P.; Fossa, P.; Capponi, S.; Geroldi, A.; Acquaviva, M.; Gulli, R.; Ciotti, P.; Manganelli, F.; Grandis, M.; Bellone, E. Clinical Features and Molecular Modelling of Novel MPZ Mutations in Demyelinating and Axonal Neuropathies. *Eur. J. Hum. Genet.* **2009**, *17*, 1129–1134, doi:10.1038/ejhg.2009.37.
154. Wrabetz, L.; Feltri, M.L.; Quattrini, A.; Imperiale, D.; Previtali, S.; D'Antonio, M.; Martini, R.; Yin, X.; Trapp, B.D.; Zhou, L.; et al. P(0) Glycoprotein Overexpression Causes Congenital Hypomyelination of Peripheral Nerves. *J. Cell Biol.* **2000**, *148*, 1021–1034, doi:10.1083/jcb.148.5.1021.
155. Bolino, A.; Muglia, M.; Conforti, F.L.; LeGuern, E.; Salih, M.A.; Georgiou, D.M.; Christodoulou, K.; Hausmanowa-Petrusewicz, I.; Mandich, P.; Schenone, A.; et al. Charcot-Marie-Tooth Type 4B Is Caused by Mutations in the Gene Encoding Myotubularin-Related Protein-2. *Nat. Genet.* **2000**, *25*, 17–19, doi:10.1038/75542.
156. Abdalla-Moady, T.; Peleg, A.; Sadeh, O.; Badarneh, K.; Fares, F. Whole-Exome Sequencing Identifies a Novel Homozygous Frameshift Mutation in the MTMR2 Gene as a Causative Mutation in a Patient with Charcot-Marie-Tooth Disease Type 4B1. *Mol. Neurobiol.* **2018**, *55*, 3546–3550, doi:10.1007/s12035-017-0588-1.
157. Bolino, A.; Bolis, A.; Previtali, S.C.; Dina, G.; Bussini, S.; Dati, G.; Amadio, S.; Del Carro, U.; Mruk, D.D.; Feltri, M.L.; et al. Disruption of Mtmr2 Produces CMT4B1-like Neuropathy with Myelin Outfolding and Impaired Spermatogenesis. *J. Cell Biol.* **2004**, *167*, 711–721, doi:10.1083/jcb.200407010.
158. Choi, B.-O.; Kang, S.H.; Hyun, Y.S.; Kanwal, S.; Park, S.W.; Koo, H.; Kim, S.-B.; Choi, Y.-C.; Yoo, J.H.; Kim, J.-W.; et al. A Complex Phenotype of Peripheral Neuropathy, Myopathy, Hoarseness, and Hearing Loss Is Linked to an Autosomal Dominant Mutation in MYH14. *Hum. Mutat.* **2011**, *32*, 669–677, doi:10.1002/humu.21488.
159. Iyadurai, S.; Arnold, W.D.; Kissel, J.T.; Ruhno, C.; Mcgovern, V.L.; Snyder, P.J.; Prior, T.W.; Roggenbuck, J.; Burghes, A.H.; Kolb, S.J. Variable Phenotypic Expression and Onset in MYH14 Distal Hereditary Motor Neuropathy Phenotype in a Large, Multigenerational North American Family. *Muscle Nerve* **2017**, *56*, 341–345, doi:10.1002/mus.25491.
160. Almutawa, W.; Smith, C.; Sabouny, R.; Smit, R.B.; Zhao, T.; Wong, R.; Lee-Glover, L.; Desrochers-Goyette, J.; Ilamathi, H.S.; Care4Rare Canada Consortium; et al. The R941L Mutation in MYH14 Disrupts Mitochondrial Fission and Associates with Peripheral Neuropathy. *EBioMedicine* **2019**, *45*, 379–392, doi:10.1016/j.ebiom.2019.06.018.
161. Tétreault, M.; Gonzalez, M.; Dicaire, M.-J.; Allard, P.; Gehring, K.; Leblanc, D.; Leclerc, N.; Schondorf, R.; Mathieu, J.; Zuchner, S.; et al. Adult-Onset Painful Axonal Polyneuropathy Caused by a Dominant NAGLU Mutation. *Brain* **2015**, *138*, 1477–1483, doi:10.1093/brain/awv074.
162. Kalaydjieva, L.; Gresham, D.; Gooding, R.; Heather, L.; Baas, F.; de Jonge, R.; Blechschmidt, K.; Angelicheva, D.; Chandler, D.; Worsley, P.; et al. N-Myc Downstream-Regulated Gene 1 Is Mutated in Hereditary Motor and Sensory Neuropathy-Lom. *Am. J. Hum. Genet.* **2000**, *67*, 47–58, doi:10.1086/302978.
163. Hunter, M.; Bernard, R.; Freitas, E.; Boyer, A.; Morar, B.; Martins, I.J.; Tournev, I.; Jordanova, A.; Guergelcheva, V.; Ishpekova, B.; et al. Mutation Screening of the N-Myc Downstream-Regulated Gene 1 (NDRG1) in Patients with Charcot-Marie-Tooth Disease. *Hum. Mutat.* **2003**, *22*, 129–135, doi:10.1002/humu.10240.
164. Okuda, T.; Higashi, Y.; Kokame, K.; Tanaka, C.; Kondoh, H.; Miyata, T. Ndrg1-Deficient Mice Exhibit a Progressive Demyelinating Disorder of Peripheral Nerves. *Mol. Cell. Biol.* **2004**, *24*, 3949–3956, doi:10.1128/mcb.24.9.3949-3956.2004.
165. Rebelo, A.P.; Abrams, A.J.; Cottenie, E.; Horga, A.; Gonzalez, M.; Bis, D.M.; Sanchez-Mejias, A.; Pinto, M.; Buglo, E.; Markel, K.; et al. Cryptic Amyloidogenic Elements in the 3' UTRs of Neurofilament Genes Trigger Axonal Neuropathy. *Am. J. Hum. Genet.* **2016**, *98*, 597–614, doi:10.1016/j.ajhg.2016.02.022.
166. Ikenberg, E.; Reilich, P.; Abicht, A.; Heller, C.; Schoser, B.; Walter, M.C. Charcot-Marie-Tooth Disease Type 2CC Due to a Frameshift Mutation of the Neurofilament Heavy Polypeptide Gene in an Austrian Family. *Neuromuscul. Disord.* **2019**, *29*, 392–397, doi:10.1016/j.nmd.2019.02.007.
167. Mersiyanova, I.V.; Perepelov, A.V.; Polyakov, A.V.; Sitnikov, V.F.; Dadali, E.L.; Oparin, R.B.; Petrin, A.N.; Evgrafov, O.V. A New Variant of Charcot-Marie-Tooth Disease Type 2 Is Probably the Result of a Mutation in the Neurofilament-Light Gene. *Am. J. Hum. Genet.* **2000**, *67*, 37–46, doi:10.1086/302962.

168. Jordanova, A.; De Jonghe, P.; Boerkoel, C.F.; Takashima, H.; De Vriendt, E.; Ceuterick, C.; Martin, J.-J.; Butler, I.J.; Mancias, P.; Papasozomenos, S.C.; et al. Mutations in the Neurofilament Light Chain Gene (NEFL) Cause Early Onset Severe Charcot-Marie-Tooth Disease. *Brain* **2003**, *126*, 590–597, doi:10.1093/brain/awg059.
169. Brownlees, J.; Ackerley, S.; Grierson, A.J.; Jacobsen, N.J.O.; Shea, K.; Anderton, B.H.; Leigh, P.N.; Shaw, C.E.; Miller, C.C.J. Charcot-Marie-Tooth Disease Neurofilament Mutations Disrupt Neurofilament Assembly and Axonal Transport. *Hum. Mol. Genet.* **2002**, *11*, 2837–2844, doi:10.1093/hmg/11.23.2837.
170. Einarsdottir, E.; Carlsson, A.; Minde, J.; Toolanen, G.; Svensson, O.; Solders, G.; Holmgren, G.; Holmberg, D.; Holmberg, M. A Mutation in the Nerve Growth Factor Beta Gene (NGFB) Causes Loss of Pain Perception. *Hum. Mol. Genet.* **2004**, *13*, 799–805, doi:10.1093/hmg/ddh096.
171. Carvalho, O.P.; Thornton, G.K.; Hertecant, J.; Houlden, H.; Nicholas, A.K.; Cox, J.J.; Rielly, M.; Al-Gazali, L.; Woods, C.G. A Novel NGF Mutation Clarifies the Molecular Mechanism and Extends the Phenotypic Spectrum of the HSAN5 Neuropathy. *J. Med. Genet.* **2011**, *48*, 131–135, doi:10.1136/jmg.2010.081455.
172. Sung, K.; Ferrari, L.F.; Yang, W.; Chung, C.; Zhao, X.; Gu, Y.; Lin, S.; Zhang, K.; Cui, B.; Pearn, M.L.; et al. Swedish Nerve Growth Factor Mutation (NGFR100W) Defines a Role for TrkA and P75NTR in Nociception. *J. Neurosci.* **2018**, *38*, 3394–3413, doi:10.1523/JNEUROSCI.1686-17.2018.
173. Ring, H.Z.; Chang, H.; Guilbot, A.; Brice, A.; LeGuern, E.; Francke, U. The Human Neuregulin-2 (NRG2) Gene: Cloning, Mapping and Evaluation as a Candidate for the Autosomal Recessive Form of Charcot-Marie-Tooth Disease Linked to 5q. *Hum Genet* **1999**, *104*, 326–332, doi:10.1007/s004390050961.
174. Mardy, S.; Miura, Y.; Endo, F.; Matsuda, I.; Sztriha, L.; Frossard, P.; Moosa, A.; Ismail, E.A.; Macaya, A.; Andria, G.; et al. Congenital Insensitivity to Pain with Anhidrosis: Novel Mutations in the TRKA (NTRK1) Gene Encoding a High-Affinity Receptor for Nerve Growth Factor. *Am. J. Hum. Genet.* **1999**, *64*, 1570–1579, doi:10.1086/302422.
175. Nam, T.-S.; Li, W.; Yoon, S.; Eom, G.H.; Kim, M.-K.; Jung, S.T.; Choi, S.-Y. Novel NTRK1 Mutations Associated with Congenital Insensitivity to Pain with Anhidrosis Verified by Functional Studies. *J. Peripher. Nerv. Syst.* **2017**, *22*, 92–99, doi:10.1111/jns.12205.
176. Shaikh, S.S.; Chen, Y.-C.; Halsall, S.-A.; Nahorski, M.S.; Omoto, K.; Young, G.T.; Phelan, A.; Woods, C.G. A Comprehensive Functional Analysis of NTRK1 Missense Mutations Causing Hereditary Sensory and Autonomic Neuropathy Type IV (HSAN IV). *Hum. Mutat.* **2017**, *38*, 55–63, doi:10.1002/humu.23123.
177. Kennerson, M.L.; Yiu, E.M.; Chuang, D.T.; Kidambi, A.; Tso, S.-C.; Ly, C.; Chaudhry, R.; Drew, A.P.; Rance, G.; Delatycki, M.B.; et al. A New Locus for X-Linked Dominant Charcot-Marie-Tooth Disease (CMTX6) Is Caused by Mutations in the Pyruvate Dehydrogenase Kinase Isoenzyme 3 (PDK3) Gene. *Hum. Mol. Genet.* **2013**, *22*, 1404–1416, doi:10.1093/hmg/dds557.
178. Kennerson, M.L.; Kim, E.J.; Siddell, A.; Kidambi, A.; Kim, S.M.; Hong, Y.B.; Hwang, S.H.; Chung, K.W.; Choi, B.-O. X-Linked Charcot-Marie-Tooth Disease Type 6 (CMTX6) Patients with a p.R158H Mutation in the Pyruvate Dehydrogenase Kinase Isoenzyme 3 Gene. *J. Peripher. Nerv. Syst.* **2016**, *21*, 45–51, doi:10.1111/jns.12160.
179. Mukherji, M.; Chien, W.; Kershaw, N.J.; Clifton, I.J.; Schofield, C.J.; Wierzbicki, A.S.; Lloyd, M.D. Structure-Function Analysis of Phytanoyl-CoA 2-Hydroxylase Mutations Causing Refsum's Disease. *Hum Mol Genet* **2001**, *10*, 1971–1982, doi:10.1093/hmg/10.18.1971.
180. Jennings, M.J.; Lochmüller, A.; Atalaia, A.; Horvath, R. Targeted Therapies for Hereditary Peripheral Neuropathies: Systematic Review and Steps Towards a "Treatabolome." *J Neuromuscul Dis* **2021**, *8*, 383–400, doi:10.3233/JND-200546.
181. Azzedine, H.; Zavadakova, P.; Planté-Bordeneuve, V.; Vaz Pato, M.; Pinto, N.; Bartesaghi, L.; Zenker, J.; Poirot, O.; Bernard-Marissal, N.; Arnaud Gouttenoire, E.; et al. PLEKHG5 Deficiency Leads to an Intermediate Form of Autosomal-Recessive Charcot-Marie-Tooth Disease. *Hum. Mol. Genet.* **2013**, *22*, 4224–4232, doi:10.1093/hmg/ddt274.
182. Kim, H.J.; Hong, Y.B.; Park, J.-M.; Choi, Y.-R.; Kim, Y.J.; Yoon, B.R.; Koo, H.; Yoo, J.H.; Kim, S.B.; Park, M.; et al. Mutations in the PLEKHG5 Gene Is Relevant with Autosomal Recessive Intermediate Charcot-Marie-Tooth Disease. *Orphanet J Rare Dis* **2013**, *8*, 104, doi:10.1186/1750-1172-8-104.

183. Gonzaga-Jauregui, C.; Harel, T.; Gambin, T.; Kousi, M.; Griffin, L.B.; Francescatto, L.; Ozes, B.; Karaca, E.; Jhangiani, S.N.; Bainbridge, M.N.; et al. Exome Sequence Analysis Suggests That Genetic Burden Contributes to Phenotypic Variability and Complex Neuropathy. *Cell Rep* **2015**, *12*, 1169–1183, doi:10.1016/j.celrep.2015.07.023.
184. Hong, Y.B.; Joo, J.; Hyun, Y.S.; Kwak, G.; Choi, Y.-R.; Yeo, H.K.; Jwa, D.H.; Kim, E.J.; Mo, W.M.; Nam, S.H.; et al. A Mutation in PMP2 Causes Dominant Demyelinating Charcot-Marie-Tooth Neuropathy. *PLoS Genet.* **2016**, *12*, e1005829, doi:10.1371/journal.pgen.1005829.
185. Lupski, J.R.; Garcia, CA; Parry, GJ Charcot-Marie-Tooth Polyneuropathy Syndrome: Clinical, Electrophysiological, and Genetic Aspects. In *Current Neurology*; Chicago Mosby-Yearbook; 1991; Vol. 11, pp. 1–15.
186. Timmerman, V.; Nelis, E.; Van Hul, W.; Nieuwenhuijsen, B.W.; Chen, K.L.; Wang, S.; Ben Othman, K.; Cullen, B.; Leach, R.J.; Hanemann, C.O. The Peripheral Myelin Protein Gene PMP-22 Is Contained within the Charcot-Marie-Tooth Disease Type 1A Duplication. *Nat. Genet.* **1992**, *1*, 171–175, doi:10.1038/ng0692-171.
187. Vance, J.M. Hereditary Motor and Sensory Neuropathies. *J. Med. Genet.* **1991**, *28*, 1–5, doi:10.1136/jmg.28.1.1.
188. Benquey, T.; Fockens, E.; Kouton, L.; Delmont, E.; Martini, N.; Levy, N.; Attarian, S.; Bonello-Palot, N. A New Point Mutation in the PMP22 Gene in a Family Suffering From Atypical HNPP. *J Neuromuscul Dis* **2020**, doi:10.3233/JND-190460.
189. Poulton, C.; Oegema, R.; Heijsman, D.; Hoogeboom, J.; Schot, R.; Stroink, H.; Willemsen, M.A.; Verheijen, F.W.; van de Spek, P.; Kremer, A.; et al. Progressive Cerebellar Atrophy and Polyneuropathy: Expanding the Spectrum of PNKP Mutations. *Neurogenetics* **2013**, *14*, 43–51, doi:10.1007/s10048-012-0351-8.
190. Djordjevic, D.; Pinard, M.; Gauthier, M.-S.; Smith-Hicks, C.; Hoffman, T.L.; Wolf, N.I.; Oegema, R.; van Binsbergen, E.; Baskin, B.; Bernard, G.; et al. De Novo Variants in POLR3B Cause Ataxia, Spasticity, and Demyelinating Neuropathy. *Am J Hum Genet* **2021**, *108*, 186–193, doi:10.1016/j.ajhg.2020.12.002.
191. Xue, Y.-Y.; Cheng, H.-L.; Dong, H.-L.; Yin, H.-M.; Yuan, Y.; Meng, L.-C.; Wu, Z.-Y.; Yu, H. A de Novo Variant of POLR3B Causes Demyelinating Charcot-Marie-Tooth Disease in a Chinese Patient: A Case Report. *BMC Neurol* **2021**, *21*, 402, doi:10.1186/s12883-021-02399-y.
192. Chen, Y.-C.; Auer-Grumbach, M.; Matsukawa, S.; Zitzelsberger, M.; Themistocleous, A.C.; Strom, T.M.; Samara, C.; Moore, A.W.; Cho, L.T.-Y.; Young, G.T.; et al. Transcriptional Regulator PRDM12 Is Essential for Human Pain Perception. *Nat. Genet.* **2015**, *47*, 803–808, doi:10.1038/ng.3308.
193. Zhang, S.; Malik Sharif, S.; Chen, Y.-C.; Valente, E.-M.; Ahmed, M.; Sheridan, E.; Bennett, C.; Woods, G. Clinical Features for Diagnosis and Management of Patients with PRDM12 Congenital Insensitivity to Pain. *J. Med. Genet.* **2016**, *53*, 533–535, doi:10.1136/jmedgenet-2015-103646.
194. Nagy, V.; Cole, T.; Van Campenhout, C.; Khoung, T.M.; Leung, C.; Vermeiren, S.; Novatchkova, M.; Wenzel, D.; Cikes, D.; Polyansky, A.A.; et al. The Evolutionarily Conserved Transcription Factor PRDM12 Controls Sensory Neuron Development and Pain Perception. *Cell Cycle* **2015**, *14*, 1799–1808, doi:10.1080/15384101.2015.1036209.
195. Kim, H.-J.; Sohn, K.-M.; Shy, M.E.; Krajewski, K.M.; Hwang, M.; Park, J.-H.; Jang, S.-Y.; Won, H.-H.; Choi, B.-O.; Hong, S.H.; et al. Mutations in PRPS1, Which Encodes the Phosphoribosyl Pyrophosphate Synthetase Enzyme Critical for Nucleotide Biosynthesis, Cause Hereditary Peripheral Neuropathy with Hearing Loss and Optic Neuropathy (Cmtx5). *Am. J. Hum. Genet.* **2007**, *81*, 552–558, doi:10.1086/519529.
196. de Brouwer, A.P.M.; van Bokhoven, H.; Nabuurs, S.B.; Arts, W.F.; Christodoulou, J.; Duley, J. PRPS1 Mutations: Four Distinct Syndromes and Potential Treatment. *Am. J. Hum. Genet.* **2010**, *86*, 506–518, doi:10.1016/j.ajhg.2010.02.024.
197. Meng, L.; Wang, K.; Lv, H.; Wang, Z.; Zhang, W.; Yuan, Y. A Novel Mutation in PRPS1 Causes X-Linked Charcot-Marie-Tooth Disease-5. *Neuropathology* **2019**, *39*, 342–347, doi:10.1111/neup.12589.
198. Delague, V.; Bareil, C.; Tuffery, S.; Bouvagnet, P.; Chouery, E.; Koussa, S.; Maisonobe, T.; Loiselet, J.; Mégarbané, A.; Claustres, M. Mapping of a New Locus for Autosomal Recessive Demyelinating Charcot-Marie-Tooth Disease to 19q13.1-13.3 in a Large Consanguineous Lebanese Family: Exclusion of MAG as a Candidate Gene. *Am. J. Hum. Genet.* **2000**, *67*, 236–243, doi:10.1086/302980.

199. Kijima, K.; Numakura, C.; Shirahata, E.; Sawaishi, Y.; Shimohata, M.; Igarashi, S.; Tanaka, T.; Hayasaka, K. Periaxin Mutation Causes Early-Onset but Slow-Progressive Charcot-Marie-Tooth Disease. *J. Hum. Genet.* **2004**, *49*, 376–379, doi:10.1007/s10038-004-0162-3.
200. Gillespie, C.S.; Sherman, D.L.; Fleetwood-Walker, S.M.; Cottrell, D.F.; Tait, S.; Garry, E.M.; Wallace, V.C.; Ure, J.; Griffiths, I.R.; Smith, A.; et al. Peripheral Demyelination and Neuropathic Pain Behavior in Periaxin-Deficient Mice. *Neuron* **2000**, *26*, 523–531, doi:10.1016/s0896-6273(00)81184-8.
201. Verhoeven, K.; De Jonghe, P.; Coen, K.; Verpoorten, N.; Auer-Grumbach, M.; Kwon, J.M.; FitzPatrick, D.; Schmedding, E.; De Vriendt, E.; Jacobs, A.; et al. Mutations in the Small GTP-Ase Late Endosomal Protein RAB7 Cause Charcot-Marie-Tooth Type 2B Neuropathy. *Am. J. Hum. Genet.* **2003**, *72*, 722–727, doi:10.1086/367847.
202. Houlden, H.; King, R.H.M.; Muddle, J.R.; Warner, T.T.; Reilly, M.M.; Orrell, R.W.; Ginsberg, L. A Novel RAB7 Mutation Associated with Ulcero-Mutilating Neuropathy. *Ann. Neurol.* **2004**, *56*, 586–590, doi:10.1002/ana.20281.
203. Janssens, K.; Goethals, S.; Atkinson, D.; Ermanoska, B.; Fransen, E.; Jordanova, A.; Auer-Grumbach, M.; Asselbergh, B.; Timmerman, V. Human Rab7 Mutation Mimics Features of Charcot-Marie-Tooth Neuropathy Type 2B in Drosophila. *Neurobiol. Dis.* **2014**, *65*, 211–219, doi:10.1016/j.nbd.2014.01.021.
204. Beetz, C.; Pieber, T.R.; Hertel, N.; Schabhüttl, M.; Fischer, C.; Trajanoski, S.; Graf, E.; Keiner, S.; Kurth, I.; Wieland, T.; et al. Exome Sequencing Identifies a REEP1 Mutation Involved in Distal Hereditary Motor Neuropathy Type V. *Am. J. Hum. Genet.* **2012**, *91*, 139–145, doi:10.1016/j.ajhg.2012.05.007.
205. Toft, A.; Birk, S.; Ballegaard, M.; Dunø, M.; Hjermind, L.E.; Nielsen, J.E.; Svenstrup, K. Peripheral Neuropathy in Hereditary Spastic Paraplegia Caused by REEP1 Variants. *J. Neurol.* **2019**, *266*, 735–744, doi:10.1007/s00415-019-09196-1.
206. Kurth, I.; Pamminger, T.; Hennings, J.C.; Soehendra, D.; Huebner, A.K.; Rotthier, A.; Baets, J.; Senderek, J.; Topaloglu, H.; Farrell, S.A.; et al. Mutations in FAM134B, Encoding a Newly Identified Golgi Protein, Cause Severe Sensory and Autonomic Neuropathy. *Nat. Genet.* **2009**, *41*, 1179–1181, doi:10.1038/ng.464.
207. Ilgaz Aydinlar, E.; Rolfs, A.; Serteser, M.; Parman, Y. Mutation in FAM134B Causing Hereditary Sensory Neuropathy with Spasticity in a Turkish Family. *Muscle Nerve* **2014**, *49*, 774–775, doi:10.1002/mus.24145.
208. Khaminets, A.; Heinrich, T.; Mari, M.; Grumati, P.; Huebner, A.K.; Akutsu, M.; Liebmann, L.; Stolz, A.; Nietzsche, S.; Koch, N.; et al. Regulation of Endoplasmic Reticulum Turnover by Selective Autophagy. *Nature* **2015**, *522*, 354–358, doi:10.1038/nature14498.
209. Nakhro, K.; Park, J.-M.; Hong, Y.B.; Park, J.H.; Nam, S.H.; Yoon, B.R.; Yoo, J.H.; Koo, H.; Jung, S.-C.; Kim, H.-L.; et al. SET Binding Factor 1 (SBF1) Mutation Causes Charcot-Marie-Tooth Disease Type 4B3. *Neurology* **2013**, *81*, 165–173, doi:10.1212/WNL.0b013e31829a3421.
210. Alazami, A.M.; Alzahrani, F.; Bohlega, S.; Alkuraya, F.S. SET Binding Factor 1 (SBF1) Mutation Causes Charcot-Marie-Tooth Disease Type 4B3. *Neurology* **2014**, *82*, 1665–1666, doi:10.1212/WNL.0000000000000331.
211. Senderek, J.; Bergmann, C.; Weber, S.; Ketelsen, U.-P.; Schorle, H.; Rudnik-Schöneborn, S.; Büttner, R.; Buchheim, E.; Zerres, K. Mutation of the SBF2 Gene, Encoding a Novel Member of the Myotubularin Family, in Charcot-Marie-Tooth Neuropathy Type 4B2/11p15. *Hum. Mol. Genet.* **2003**, *12*, 349–356, doi:10.1093/hmg/ddg030.
212. Azzedine, H.; Bolino, A.; Taïeb, T.; Birouk, N.; Di Duca, M.; Bouhouche, A.; Benamou, S.; Mrabet, A.; Hammadouche, T.; Chkili, T.; et al. Mutations in MTMR13, a New Pseudophosphatase Homologue of MTMR2 and Sbf1, in Two Families with an Autosomal Recessive Demyelinating Form of Charcot-Marie-Tooth Disease Associated with Early-Onset Glaucoma. *Am. J. Hum. Genet.* **2003**, *72*, 1141–1153, doi:10.1086/375034.
213. Tersar, K.; Boentert, M.; Berger, P.; Bonneick, S.; Wessig, C.; Toyka, K.V.; Young, P.; Suter, U. Mtmr13/Sbf2-Deficient Mice: An Animal Model for CMT4B2. *Hum. Mol. Genet.* **2007**, *16*, 2991–3001, doi:10.1093/hmg/ddm257.
214. Faber, C.G.; Lauria, G.; Merkies, I.S.J.; Cheng, X.; Han, C.; Ahn, H.-S.; Persson, A.-K.; Hoeijmakers, J.G.J.; Gerrits, M.M.; Pierro, T.; et al. Gain-of-Function Nav1.8 Mutations in Painful Neuropathy. *Proc. Natl. Acad. Sci. U.S.A.* **2012**, *109*, 19444–19449, doi:10.1073/pnas.1216080109.

215. Duan, G.; Sun, J.; Li, N.; Zheng, H.; Guo, S.; Zhang, Y.; Wang, Q.; Ying, Y.; Zhang, M.; Huang, P.; et al. A Variant in the SCN10A Enhancer May Affect Human Mechanical Pain Sensitivity. *Mol Pain* **2018**, *14*, 1744806918763275, doi:10.1177/1744806918763275.
216. Leipold, E.; Liebmann, L.; Korenke, G.C.; Heinrich, T.; Giesselmann, S.; Baets, J.; Ebbinghaus, M.; Goral, R.O.; Stödberg, T.; Hennings, J.C.; et al. A de Novo Gain-of-Function Mutation in SCN11A Causes Loss of Pain Perception. *Nat. Genet.* **2013**, *45*, 1399–1404, doi:10.1038/ng.2767.
217. Woods, C.G.; Babiker, M.O.E.; Horrocks, I.; Tolmie, J.; Kurth, I. The Phenotype of Congenital Insensitivity to Pain Due to the NaV1.9 Variant p.L811P. *Eur. J. Hum. Genet.* **2015**, *23*, 561–563, doi:10.1038/ejhg.2014.166.
218. Priest, B.T.; Murphy, B.A.; Lindia, J.A.; Diaz, C.; Abbadie, C.; Ritter, A.M.; Liberator, P.; Iyer, L.M.; Kash, S.F.; Kohler, M.G.; et al. Contribution of the Tetrodotoxin-Resistant Voltage-Gated Sodium Channel NaV1.9 to Sensory Transmission and Nociceptive Behavior. *Proc. Natl. Acad. Sci. U.S.A.* **2005**, *102*, 9382–9387, doi:10.1073/pnas.0501549102.
219. Staud, R.; Price, D.D.; Janicke, D.; Andrade, E.; Hadjipanayis, A.G.; Eaton, W.T.; Kaplan, L.; Wallace, M.R. Two Novel Mutations of SCN9A (Nav1.7) Are Associated with Partial Congenital Insensitivity to Pain. *Eur J Pain* **2011**, *15*, 223–230, doi:10.1016/j.ejpain.2010.07.003.
220. Yuan, J.; Matsuura, E.; Higuchi, Y.; Hashiguchi, A.; Nakamura, T.; Nozuma, S.; Sakiyama, Y.; Yoshimura, A.; Izumo, S.; Takashima, H. Hereditary Sensory and Autonomic Neuropathy Type IID Caused by an SCN9A Mutation. *Neurology* **2013**, *80*, 1641–1649, doi:10.1212/WNL.0b013e3182904fdd.
221. Duquette, A.; Roddier, K.; McNabb-Baltar, J.; Gosselin, I.; St-Denis, A.; Dicaire, M.-J.; Loisel, L.; Labuda, D.; Marchand, L.; Mathieu, J.; et al. Mutations in Senataxin Responsible for Quebec Cluster of Ataxia with Neuropathy. *Ann. Neurol.* **2005**, *57*, 408–414, doi:10.1002/ana.20408.
222. Moreira, M.-C.; Klur, S.; Watanabe, M.; Németh, A.H.; Le Ber, I.; Moniz, J.-C.; Tranchant, C.; Aubourg, P.; Tazir, M.; Schöls, L.; et al. Senataxin, the Ortholog of a Yeast RNA Helicase, Is Mutant in Ataxia-Ocular Apraxia 2. *Nat. Genet.* **2004**, *36*, 225–227, doi:10.1038/ng1303.
223. Airoldi, G.; Guidarelli, A.; Cantoni, O.; Panzeri, C.; Vantaggiato, C.; Bonato, S.; Grazia D'Angelo, M.; Falcone, S.; De Palma, C.; Tonelli, A.; et al. Characterization of Two Novel SETX Mutations in AOA2 Patients Reveals Aspects of the Pathophysiological Role of Senataxin. *Neurogenetics* **2010**, *11*, 91–100, doi:10.1007/s10048-009-0206-0.
224. Senderek, J.; Bergmann, C.; Stendel, C.; Kirfel, J.; Verpoorten, N.; De Jonghe, P.; Timmerman, V.; Chrast, R.; Verheijen, M.H.G.; Lemke, G.; et al. Mutations in a Gene Encoding a Novel SH3/TPR Domain Protein Cause Autosomal Recessive Charcot-Marie-Tooth Type 4C Neuropathy. *Am. J. Hum. Genet.* **2003**, *73*, 1106–1119, doi:10.1086/379525.
225. Gooding, R.; Colomer, J.; King, R.; Angelicheva, D.; Marns, L.; Parman, Y.; Chandler, D.; Bertranpetit, J.; Kalaydjieva, L. A Novel Gypsy Founder Mutation, p.Arg1109X in the CMT4C Gene, Causes Variable Peripheral Neuropathy Phenotypes. *J. Med. Genet.* **2005**, *42*, e69, doi:10.1136/jmg.2005.034132.
226. Arnaud, E.; Zenker, J.; de Preux Charles, A.-S.; Stendel, C.; Roos, A.; Médard, J.-J.; Tricaud, N.; Kleine, H.; Luscher, B.; Weis, J.; et al. SH3TC2/KIAA1985 Protein Is Required for Proper Myelination and the Integrity of the Node of Ranvier in the Peripheral Nervous System. *Proc. Natl. Acad. Sci. U.S.A.* **2009**, *106*, 17528–17533, doi:10.1073/pnas.0905523106.
227. Li, X.; Hu, Z.; Liu, L.; Xie, Y.; Zhan, Y.; Zi, X.; Wang, J.; Wu, L.; Xia, K.; Tang, B.; et al. A SIGMAR1 Splice-Site Mutation Causes Distal Hereditary Motor Neuropathy. *Neurology* **2015**, *84*, 2430–2437, doi:10.1212/WNL.0000000000001680.
228. Lee, J.J.Y.; van Karnebeek, C.D.M.; Drögemoller, B.; Shyr, C.; Tarailo-Graovac, M.; Eydoux, P.; Ross, C.J.; Wasserman, W.W.; Björnson, B.; Wu, J.K. Further Validation of the SIGMAR1 c.151+1G>T Mutation as Cause of Distal Hereditary Motor Neuropathy. *Child Neurol Open* **2016**, *3*, 2329048X16669912, doi:10.1177/2329048X16669912.
229. Gregianin, E.; Pallafacchina, G.; Zanin, S.; Crippa, V.; Rusmini, P.; Poletti, A.; Fang, M.; Li, Z.; Diano, L.; Petrucci, A.; et al. Loss-of-Function Mutations in the SIGMAR1 Gene Cause Distal Hereditary Motor Neuropathy by Impairing ER-Mitochondria Tethering and Ca2+ Signalling. *Hum. Mol. Genet.* **2016**, *25*, 3741–3753, doi:10.1093/hmg/ddw220.

230. Abrams, A.J.; Hufnagel, R.B.; Rebelo, A.; Zanna, C.; Patel, N.; Gonzalez, M.A.; Campeanu, I.J.; Griffin, L.B.; Groenewald, S.; Strickland, A.V.; et al. Mutations in SLC25A46, Encoding a UGO1-like Protein, Cause an Optic Atrophy Spectrum Disorder. *Nat Genet* **2015**, *47*, 926–932, doi:10.1038/ng.3354.
231. Charlesworth, G.; Balint, B.; Mencacci, N.E.; Carr, L.; Wood, N.W.; Bhatia, K.P. SLC25A46 Mutations Underlie Progressive Myoclonic Ataxia with Optic Atrophy and Neuropathy. *Mov Disord* **2016**, *31*, 1249–1251, doi:10.1002/mds.26716.
232. Barwick, K.E.S.; Wright, J.; Al-Turki, S.; McEntagart, M.M.; Nair, A.; Chioza, B.; Al-Memar, A.; Modarres, H.; Reilly, M.M.; Dick, K.J.; et al. Defective Presynaptic Choline Transport Underlies Hereditary Motor Neuropathy. *Am. J. Hum. Genet.* **2012**, *91*, 1103–1107, doi:10.1016/j.ajhg.2012.09.019.
233. Hamanaka, K.; Takahashi, K.; Miyatake, S.; Mitsuhashi, S.; Hamanoue, H.; Miyaji, Y.; Fukai, R.; Doi, H.; Fujita, A.; Imagawa, E.; et al. Confirmation of SLC5A7-Related Distal Hereditary Motor Neuropathy 7 in a Family Outside Wales. *Clin. Genet.* **2018**, *94*, 274–275, doi:10.1111/cge.13369.
234. Song, G.J.; Gupta, D.P.; Rahman, M.H.; Park, H.T.; Al Ghouleh, I.; Bisello, A.; Lee, M.-G.; Park, J.-Y.; Park, H.H.; Jun, J.H.; et al. Loss-of-Function of EBP50 Is a New Cause of Hereditary Peripheral Neuropathy: EBP50 Functions in Peripheral Nerve System. *Glia* **2020**, *68*, 1794–1809, doi:10.1002/glia.23805.
235. Shaw, P.J.; Tomkins, J.; Slade, J.Y.; Usher, P.; Curtis, A.; Bushby, K.; Ince, P.G. CNS Tissue Cu/Zn Superoxide Dismutase (SOD1) Mutations in Motor Neurone Disease (MND). *Neuroreport* **1997**, *8*, 3923–3927, doi:10.1097/00001756-199712220-00016.
236. Cortese, A.; Zhu, Y.; Rebelo, A.P.; Negri, S.; Courel, S.; Abreu, L.; Bacon, C.J.; Bai, Y.; Bis-Brewer, D.M.; Bugiardini, E.; et al. Biallelic Mutations in SORD Cause a Common and Potentially Treatable Hereditary Neuropathy with Implications for Diabetes. *Nat Genet* **2020**, *52*, 473–481, doi:10.1038/s41588-020-0615-4.
237. Dong, H.-L.; Li, J.-Q.; Liu, G.-L.; Yu, H.; Wu, Z.-Y. Biallelic SORD Pathogenic Variants Cause Chinese Patients with Distal Hereditary Motor Neuropathy. *NPJ Genom Med* **2021**, *6*, 1, doi:10.1038/s41525-020-00165-6.
238. Montecchiani, C.; Pedace, L.; Lo Giudice, T.; Casella, A.; Mearini, M.; Gaudiello, F.; Pedroso, J.L.; Terracciano, C.; Caltagirone, C.; Massa, R.; et al. ALS5/SPG11/KIAA1840 Mutations Cause Autosomal Recessive Axonal Charcot-Marie-Tooth Disease. *Brain* **2016**, *139*, 73–85, doi:10.1093/brain/awv320.
239. Manole, A.; Chelban, V.; Haridy, N.A.; Hamed, S.A.; Berardo, A.; Reilly, M.M.; Houlden, H. Severe Axonal Neuropathy Is a Late Manifestation of SPG11. *J. Neurol.* **2016**, *263*, 2278–2286, doi:10.1007/s00415-016-8254-5.
240. Branchu, J.; Boutry, M.; Sourd, L.; Depp, M.; Leone, C.; Corriger, A.; Vallucci, M.; Esteves, T.; Matusiak, R.; Dumont, M.; et al. Loss of Spatacsin Function Alters Lysosomal Lipid Clearance Leading to Upper and Lower Motor Neuron Degeneration. *Neurobiol. Dis.* **2017**, *102*, 21–37, doi:10.1016/j.nbd.2017.02.007.
241. Winner, B.; Uyanik, G.; Gross, C.; Lange, M.; Schulte-Mattler, W.; Schuierer, G.; Marienhagen, J.; Hehr, U.; Winkler, J. Clinical Progression and Genetic Analysis in Hereditary Spastic Paraplegia with Thin Corpus Callosum in Spastic Gait Gene 11 (SPG11). *Arch Neurol* **2004**, *61*, 117–121, doi:10.1001/archneur.61.1.117.
242. Beijer, D.; Deconinck, T.; De Bleecker, J.L.; Dotti, M.T.; Malandrini, A.; Urtizberea, J.A.; Zulaica, M.; López de Munain, A.; Asselbergh, B.; De Jonghe, P.; et al. Nonsense Mutations in Alpha-II Spectrin in Three Families with Juvenile Onset Hereditary Motor Neuropathy. *Brain* **2019**, *142*, 2605–2616, doi:10.1093/brain/awz216.
243. Ylikallio, E.; Ritari, N.; Sainio, M.; Toppila, J.; Kivirikko, S.; Tyynismaa, H.; Auranen, M.; Isohanni, P. De Novo SPTAN1 Mutation in Axonal Sensorimotor Neuropathy and Developmental Disorder. *Brain* **2020**, *143*, e104, doi:10.1093/brain/awaa344.
244. Dawkins, J.L.; Hulme, D.J.; Brahmbhatt, S.B.; Auer-Grumbach, M.; Nicholson, G.A. Mutations in SPTLC1, Encoding Serine Palmitoyltransferase, Long Chain Base Subunit-1, Cause Hereditary Sensory Neuropathy Type I. *Nat. Genet.* **2001**, *27*, 309–312, doi:10.1038/85879.
245. Bejaoui, K.; Wu, C.; Scheffler, M.D.; Haan, G.; Ashby, P.; Wu, L.; de Jong, P.; Brown, R.H. SPTLC1 Is Mutated in Hereditary Sensory Neuropathy, Type 1. *Nat. Genet.* **2001**, *27*, 261–262, doi:10.1038/85817.

246. McCampbell, A.; Truong, D.; Broom, D.C.; Allchorne, A.; Gable, K.; Cutler, R.G.; Mattson, M.P.; Woolf, C.J.; Frosch, M.P.; Harmon, J.M.; et al. Mutant SPTLC1 Dominantly Inhibits Serine Palmitoyltransferase Activity in Vivo and Confers an Age-Dependent Neuropathy. *Hum. Mol. Genet.* **2005**, *14*, 3507–3521, doi:10.1093/hmg/ddi380.
247. Rotthier, A.; Auer-Grumbach, M.; Janssens, K.; Baets, J.; Penno, A.; Almeida-Souza, L.; Van Hoof, K.; Jacobs, A.; De Vriendt, E.; Schlotter-Weigel, B.; et al. Mutations in the SPTLC2 Subunit of Serine Palmitoyltransferase Cause Hereditary Sensory and Autonomic Neuropathy Type I. *Am. J. Hum. Genet.* **2010**, *87*, 513–522, doi:10.1016/j.ajhg.2010.09.010.
248. Murphy, S.M.; Ernst, D.; Wei, Y.; Laurà, M.; Liu, Y.-T.; Polke, J.; Blake, J.; Winer, J.; Houlden, H.; Hornemann, T.; et al. Hereditary Sensory and Autonomic Neuropathy Type 1 (HSANI) Caused by a Novel Mutation in SPTLC2. *Neurology* **2013**, *80*, 2106–2111, doi:10.1212/WNL.0b013e318295d789.
249. Echaniz-Laguna, A.; Ghezzi, D.; Chassagne, M.; Mayençon, M.; Padet, S.; Melchionda, L.; Rouvet, I.; Lannes, B.; Bozon, D.; Latour, P.; et al. SURF1 Deficiency Causes Demyelinating Charcot-Marie-Tooth Disease. *Neurology* **2013**, *81*, 1523–1530, doi:10.1212/WNL.0b013e3182a4a518.
250. Whittaker, R.G.; Herrmann, D.N.; Bansagi, B.; Hasan, B.A.S.; Lofra, R.M.; Logigian, E.L.; Sowden, J.E.; Almodovar, J.L.; Littleton, J.T.; Zuchner, S.; et al. Electrophysiologic Features of SYT2 Mutations Causing a Treatable Neuromuscular Syndrome. *Neurology* **2015**, *85*, 1964–1971, doi:10.1212/WNL.0000000000002185.
251. Montes-Chinea, N.I.; Guan, Z.; Coutts, M.; Vidal, C.; Courel, S.; Rebelo, A.P.; Abreu, L.; Zuchner, S.; Littleton, J.T.; Saporta, M.A. Identification of a New SYT2 Variant Validates an Unusual Distal Motor Neuropathy Phenotype. *Neurol Genet* **2018**, *4*, e282, doi:10.1212/NXG.0000000000000282.
252. Heimer, G.; Oz-Levi, D.; Eyal, E.; Edvardson, S.; Nissenkorn, A.; Ruzzo, E.K.; Szeinberg, A.; Maayan, C.; Mai-Zahav, M.; Efrati, O.; et al. TECPR2 Mutations Cause a New Subtype of Familial Dysautonomia like Hereditary Sensory Autonomic Neuropathy with Intellectual Disability. *Eur J Paediatr Neurol* **2016**, *20*, 69–79, doi:10.1016/j.ejpn.2015.10.003.
253. Neuser, S.; Brechmann, B.; Heimer, G.; Brösse, I.; Schubert, S.; O'Grady, L.; Zech, M.; Srivastava, S.; Sweetser, D.A.; Dincer, Y.; et al. Clinical, Neuroimaging, and Molecular Spectrum of TECPR2-Associated Hereditary Sensory and Autonomic Neuropathy with Intellectual Disability. *Hum Mutat* **2021**, *42*, 762–776, doi:10.1002/humu.24206.
254. Tamim-Yecheskel, B.-C.; Fraiberg, M.; Kokabi, K.; Freud, S.; Shatz, O.; Marvaldi, L.; Subic, N.; Brenner, O.; Tsoory, M.; Eilam-Altstadter, R.; et al. A Tecpr2 Knockout Mouse Exhibits Age-Dependent Neuroaxonal Dystrophy Associated with Autophagosome Accumulation. *Autophagy* **2021**, *17*, 3082–3095, doi:10.1080/15548627.2020.1852724.
255. Ishiura, H.; Sako, W.; Yoshida, M.; Kawarai, T.; Tanabe, O.; Goto, J.; Takahashi, Y.; Date, H.; Mitsui, J.; Ahsan, B.; et al. The TRK-Fused Gene Is Mutated in Hereditary Motor and Sensory Neuropathy with Proximal Dominant Involvement. *Am. J. Hum. Genet.* **2012**, *91*, 320–329, doi:10.1016/j.ajhg.2012.07.014.
256. Lee, S.-S.; Lee, H.J.; Park, J.-M.; Hong, Y.B.; Park, K.-D.; Yoo, J.H.; Koo, H.; Jung, S.-C.; Park, H.S.; Lee, J.H.; et al. Proximal Dominant Hereditary Motor and Sensory Neuropathy with Proximal Dominance Association with Mutation in the TRK-Fused Gene. *JAMA Neurol* **2013**, *70*, 607–615, doi:10.1001/jamaneurol.2013.1250.
257. Tsai, P.-C.; Huang, Y.-H.; Guo, Y.-C.; Wu, H.-T.; Lin, K.-P.; Tsai, Y.-S.; Liao, Y.-C.; Liu, Y.-T.; Liu, T.-T.; Kao, L.-S.; et al. A Novel TFG Mutation Causes Charcot-Marie-Tooth Disease Type 2 and Impairs TFG Function. *Neurology* **2014**, *83*, 903–912, doi:10.1212/WNL.0000000000000758.
258. Ylikallio, E.; Pöyhönen, R.; Zimon, M.; De Vriendt, E.; Hilander, T.; Paetau, A.; Jordanova, A.; Lönnqvist, T.; Tyynismaa, H. Deficiency of the E3 Ubiquitin Ligase TRIM2 in Early-Onset Axonal Neuropathy. *Hum. Mol. Genet.* **2013**, *22*, 2975–2983, doi:10.1093/hmg/ddt149.
259. Pehlivan, D.; Coban Akdemir, Z.; Karaca, E.; Bayram, Y.; Jhangiani, S.; Yildiz, E.P.; Muzny, D.; Uluc, K.; Gibbs, R.A.; Baylor-Hopkins Center for Mendelian Genomics; et al. Exome Sequencing Reveals Homozygous TRIM2 Mutation in a Patient with Early Onset CMT and Bilateral Vocal Cord Paralysis. *Hum. Genet.* **2015**, *134*, 671–673, doi:10.1007/s00439-015-1548-3.

260. Balastik, M.; Ferraguti, F.; Pires-da Silva, A.; Lee, T.H.; Alvarez-Bolado, G.; Lu, K.P.; Gruss, P. Deficiency in Ubiquitin Ligase TRIM2 Causes Accumulation of Neurofilament Light Chain and Neurodegeneration. *Proc. Natl. Acad. Sci. U.S.A.* **2008**, *105*, 12016–12021, doi:10.1073/pnas.0802261105.
261. Auer-Grumbach, M.; Olschewski, A.; Papić, L.; Kremer, H.; McEntagart, M.E.; Uhrig, S.; Fischer, C.; Fröhlich, E.; Bálint, Z.; Tang, B.; et al. Alterations in the Ankyrin Domain of TRPV4 Cause Congenital Distal SMA, Scapuloperoneal SMA and HMSN2C. *Nat. Genet.* **2010**, *42*, 160–164, doi:10.1038/ng.508.
262. Deng, H.-X.; Klein, C.J.; Yan, J.; Shi, Y.; Wu, Y.; Fecto, F.; Yau, H.-J.; Yang, Y.; Zhai, H.; Siddique, N.; et al. Scapuloperoneal Spinal Muscular Atrophy and CMT2C Are Allelic Disorders Caused by Alterations in TRPV4. *Nat. Genet.* **2010**, *42*, 165–169, doi:10.1038/ng.509.
263. Klein, C.J.; Shi, Y.; Fecto, F.; Donaghy, M.; Nicholson, G.; McEntagart, M.E.; Crosby, A.H.; Wu, Y.; Lou, H.; McEvoy, K.M.; et al. TRPV4 Mutations and Cytotoxic Hypercalcemia in Axonal Charcot-Marie-Tooth Neuropathies. *Neurology* **2011**, *76*, 887–894, doi:10.1212/WNL.0b013e31820f2de3.
264. Saraiva, M.J. Transthyretin Mutations in Hyperthyroxinemia and Amyloid Diseases. *Hum Mutat* **2001**, *17*, 493–503, doi:10.1002/humu.1132.
265. Benson, M.D. Inherited Amyloidosis. *J Med Genet* **1991**, *28*, 73–78, doi:10.1136/jmg.28.2.73.
266. Sekijima, Y.; Wiseman, R.L.; Matteson, J.; Hammarström, P.; Miller, S.R.; Sawkar, A.R.; Balch, W.E.; Kelly, J.W. The Biological and Chemical Basis for Tissue-Selective Amyloid Disease. *Cell* **2005**, *121*, 73–85, doi:10.1016/j.cell.2005.01.018.
267. Nishimura, A.L.; Mitne-Neto, M.; Silva, H.C.A.; Richieri-Costa, A.; Middleton, S.; Cascio, D.; Kok, F.; Oliveira, J.R.M.; Gillingwater, T.; Webb, J.; et al. A Mutation in the Vesicle-Trafficking Protein VAPB Causes Late-Onset Spinal Muscular Atrophy and Amyotrophic Lateral Sclerosis. *Am. J. Hum. Genet.* **2004**, *75*, 822–831, doi:10.1086/425287.
268. Chen, H.-J.; Anagnostou, G.; Chai, A.; Withers, J.; Morris, A.; Adhikaree, J.; Pennetta, G.; de Belleroche, J.S. Characterization of the Properties of a Novel Mutation in VAPB in Familial Amyotrophic Lateral Sclerosis. *J. Biol. Chem.* **2010**, *285*, 40266–40281, doi:10.1074/jbc.M110.161398.
269. Larroquette, F.; Seto, L.; Gaub, P.L.; Kamal, B.; Wallis, D.; Larivière, R.; Vallée, J.; Robitaille, R.; Tsuda, H. Vapb/Amyotrophic Lateral Sclerosis 8 Knock-in Mice Display Slowly Progressive Motor Behavior Defects Accompanying ER Stress and Autophagic Response. *Hum. Mol. Genet.* **2015**, *24*, 6515–6529, doi:10.1093/hmg/ddv360.
270. Gonzalez, M.A.; Feely, S.M.; Speziani, F.; Strickland, A.V.; Danzi, M.; Bacon, C.; Lee, Y.; Chou, T.-F.; Blanton, S.H.; Weihl, C.C.; et al. A Novel Mutation in VCP Causes Charcot-Marie-Tooth Type 2 Disease. *Brain* **2014**, *137*, 2897–2902, doi:10.1093/brain/awu224.
271. Jerath, N.U.; Crockett, C.D.; Moore, S.A.; Shy, M.E.; Weihl, C.C.; Chou, T.-F.; Grider, T.; Gonzalez, M.A.; Zuchner, S.; Swenson, A. Rare Manifestation of a c.290 C>T, p.Gly97Glu VCP Mutation. *Case Rep Genet* **2015**, *2015*, 239167, doi:10.1155/2015/239167.
272. Gonzaga-Jauregui, C.; Lotze, T.; Jamal, L.; Penney, S.; Campbell, I.M.; Pehlivan, D.; Hunter, J.V.; Woodbury, S.L.; Raymond, G.; Adesina, A.M.; et al. Mutations in VRK1 Associated with Complex Motor and Sensory Axonal Neuropathy plus Microcephaly. *JAMA Neurol* **2013**, *70*, 1491–1498, doi:10.1001/jamaneurol.2013.4598.
273. Feng, S.-Y.; Li, L.-Y.; Feng, S.-M.; Zou, Z.-Y. A Novel VRK1 Mutation Associated with Recessive Distal Hereditary Motor Neuropathy. *Ann Clin Transl Neurol* **2019**, *6*, 401–405, doi:10.1002/acn3.701.
274. El-Bazzal, L.; Rihan, K.; Bernard-Marissal, N.; Castro, C.; Chouery-Khoury, E.; Desvignes, J.-P.; Atkinson, A.; Bertaux, K.; Koussa, S.; Lévy, N.; et al. Loss of Cajal Bodies in Motor Neurons from Patients with Novel Mutations in VRK1. *Hum. Mol. Genet.* **2019**, *28*, 2378–2394, doi:10.1093/hmg/ddz060.
275. Bos, R.; Rihan, K.; Quintana, P.; El-Bazzal, L.; Bernard-Marissal, N.; Da Silva, N.; Jabbour, R.; Mégarbané, A.; Bartoli, M.; Brocard, F.; et al. Altered Action Potential Waveform and Shorter Axonal Initial Segment in HiPSC-Derived Motor Neurons with Mutations in VRK1. *Neurobiol Dis* **2022**, *164*, 105609, doi:10.1016/j.nbd.2021.105609.
276. Tsai, P.-C.; Soong, B.-W.; Mademan, I.; Huang, Y.-H.; Liu, C.-R.; Hsiao, C.-T.; Wu, H.-T.; Liu, T.-T.; Liu, Y.-T.; Tseng, Y.-T.; et al. A Recurrent WARS Mutation Is a Novel Cause of Autosomal Dominant Distal Hereditary Motor Neuropathy. *Brain* **2017**, *140*, 1252–1266, doi:10.1093/brain/awx058.

277. Wang, B.; Li, X.; Huang, S.; Zhao, H.; Liu, J.; Hu, Z.; Lin, Z.; Liu, L.; Xie, Y.; Jin, Q.; et al. A Novel WARS Mutation (p.Asp314Gly) Identified in a Chinese Distal Hereditary Motor Neuropathy Family. *Clin. Genet.* **2019**, *96*, 176–182, doi:10.1111/cge.13563.
278. Lafreniere, R.G.; MacDonald, M.L.E.; Dube, M.-P.; MacFarlane, J.; O'Driscoll, M.; Brais, B.; Meilleur, S.; Brinkman, R.R.; Dadivas, O.; Pape, T.; et al. Identification of a Novel Gene (HSN2) Causing Hereditary Sensory and Autonomic Neuropathy Type II through the Study of Canadian Genetic Isolates. *Am. J. Hum. Genet.* **2004**, *74*, 1064–1073, doi:10.1086/420795.
279. Roddier, K.; Thomas, T.; Marleau, G.; Gagnon, A.M.; Dicaire, M.J.; St-Denis, A.; Gosselin, I.; Sarrazin, A.M.; Larbrisseau, A.; Lambert, M.; et al. Two Mutations in the HSN2 Gene Explain the High Prevalence of HSAN2 in French Canadians. *Neurology* **2005**, *64*, 1762–1767, doi:10.1212/01.WNL.0000161849.29944.43.
280. Bercier, V.; Brustein, E.; Liao, M.; Dion, P.A.; Lafrenière, R.G.; Rouleau, G.A.; Drapeau, P. WNK1/HSN2 Mutation in Human Peripheral Neuropathy Deregulates KCC2 Expression and Posterior Lateral Line Development in Zebrafish (Danio Rerio). *PLoS Genet.* **2013**, *9*, e1003124, doi:10.1371/journal.pgen.1003124.
281. Jordanova, A.; Irobi, J.; Thomas, F.P.; Van Dijck, P.; Meerschaert, K.; Dewil, M.; Dierick, I.; Jacobs, A.; De Vriendt, E.; Guergueltcheva, V.; et al. Disrupted Function and Axonal Distribution of Mutant Tyrosyl-TRNA Synthetase in Dominant Intermediate Charcot-Marie-Tooth Neuropathy. *Nat. Genet.* **2006**, *38*, 197–202, doi:10.1038/ng1727.
282. Storkebaum, E.; Leitão-Gonçalves, R.; Godenschwege, T.; Nangle, L.; Mejia, M.; Bosmans, I.; Ooms, T.; Jacobs, A.; Van Dijck, P.; Yang, X.-L.; et al. Dominant Mutations in the Tyrosyl-TRNA Synthetase Gene Recapitulate in Drosophila Features of Human Charcot-Marie-Tooth Neuropathy. *Proc. Natl. Acad. Sci. U.S.A.* **2009**, *106*, 11782–11787, doi:10.1073/pnas.0905339106.
